# Linkage of alterations in systemic iron homeostasis to patients’ outcome in sepsis: a prospective study

**DOI:** 10.1186/s40560-020-00495-8

**Published:** 2020-10-01

**Authors:** Anna Brandtner, Piotr Tymoszuk, Manfred Nairz, Georg F. Lehner, Gernot Fritsche, Anja Vales, Andreas Falkner, Harald Schennach, Igor Theurl, Michael Joannidis, Günter Weiss, Christa Pfeifhofer-Obermair

**Affiliations:** 1 Division of Intensive Care and Emergency Medicine, Department of Internal Medicine I, Medical University of Innsbruck, Innsbruck, Austria; 2 Department of Internal Medicine II, Medical University of Innsbruck, Anichstr. 35, Innsbruck, Austria; 3 Christian Doppler Laboratory for Iron Metabolism and Anemia Research, Medical University of Innsbruck, Innsbruck, Austria; 4 Central Institute for Blood Transfusion and Immunology, Innsbruck, Austria

**Keywords:** Ferritin, Infection, SOFA score, Transferrin saturation, Transferrin

## Abstract

**Background:**

Sepsis, a dysregulated host response following infection, is associated with massive immune activation and high mortality rates. There is still a need to define further risk factors and laboratory parameters predicting the clinical course. Iron metabolism is regulated by both, the body’s iron status and the immune response. Iron itself is required for erythropoiesis but also for many cellular and metabolic functions. Moreover, iron availability is a critical determinant in infections because it is an essential nutrient for most microbes but also impacts on immune function and intravascular oxidative stress. Herein, we used a prospective study design to investigate the putative impact of serum iron parameters on the outcome of sepsis.

**Methods:**

Serum markers of iron metabolism were measured in a prospective cohort of 61 patients (37 males, 24 females) with sepsis defined by Sepsis-3 criteria in a medical intensive care unit (ICU) and compared between survivors and non-survivors. Regulation of iron parameters in patients stratified by focus of infection and co-medication as well as association of the markers with sepsis severity scores and survival were investigated with linear and logistic regression corrected for sex and age effects.

**Results:**

Positive correlations of increased serum iron and ferritin concentrations upon ICU admission with the severity of organ failure (SOFA score) and with mortality were observed. Moreover, high TF-Sat, elevated ferritin and serum iron levels and low transferrin concentrations were associated with reduced survival. A logistic regression model consisting of SOFA and transferrin saturation (SOFA–TF-Sat) had the best predictive power for survival in septic ICU patients. Of note, administration of blood transfusions prior to ICU admission resulted in increased TF-Sat and reduced survival of septic patients.

**Conclusions:**

Our study could show an important impact of serum iron parameters on the outcome of sepsis. Furthermore, we identified transferrin saturation as a stand-alone predictor of sepsis survival and as a parameter of iron metabolism which may in a combined model improve the prediction power of the SOFA score.

**Trial registration:**

The study was carried out in accordance with the recommendations of the Declaration of Helsinki on biomedical research. The study was approved by the institutional ethics review board of the Medical University Innsbruck (study AN2013-0006).

## Background

Sepsis is a manifestation of infections that is characterized by life-threatening organ dysfunction considered to be caused by a dysregulated host response [1]. Each year, 19 million individuals are diagnosed with sepsis. More than two thirds of patients survive, but one third of sepsis patients die within the first year and 40% need to be rehospitalized within the first 90 days after discharge [2]. A complex interplay of multiple physiological and pathophysiological processes contributes to organ dysfunction, the central feature of this syndrome. Among those, iron metabolism is one of the systems which are heavily affected by severe inflammation [3, 4].

Iron is a central compound of the proteins hemoglobin and myoglobin where it is responsible for the binding of oxygen. Iron is crucial for donating and accepting electrons in processes involved in mitochondrial respiration, citric acid cycle, DNA synthesis or hormone production. A balanced body iron homeostasis is essential for basic metabolic functions [1, 5]. After phagocytosis of erythrocytes by macrophages and degradation of heme, iron leaves the cytosol through the only known iron-exporter ferroportin-1 (Fpn-1), which is tightly regulated by the hormone hepcidin [6]. Iron is transported within the circulation following binding to transferrin (Tf) and taken up by metabolically active cells via transferrin receptor (TfR-1) [5]. Excess iron is stored within ferritin (Ft) to avoid toxicity of pro-oxidative labile iron (Additional file 1).

The upregulation of hepcidin in response to inflammatory processes blocks iron recycling from macrophages and dietary uptake in the duodenum thereby resulting in the reduction of circulating iron levels [7]. Collectively, these processes are the hallmarks of functional iron deficiency. As both the host and several invading microbes are highly dependent on iron availability, this reduction of circulating iron levels in the course of infection is considered as an important part of host defense as strategy that limits pathogen growth [8, 9] (Additional file 1). Moreover, iron has multiple regulatory effects on immune function, among others blocking anti-microbial immune pathways or stimulating the production of the anti-inflammatory cytokine IL-10 [10]. As a consequence, this functional iron deficiency leads to anemia of inflammation (AI) or anemia of chronic disease (ACD) which is also found in critically ill patients [11, 12] (Additional file 1). ACD is diagnosed upon the presence of reduced hemoglobin levels, increased concentrations of inflammation markers, low circulating iron levels, a reduced saturation of transferrin, along with normal or increased concentrations of ferritin [13]. In critically ill patients, serum ferritin levels are upregulated by cytokines yet independent of iron availability and therefore are not a reliable marker during inflammation [14]. Recently, the soluble TfR (sTfR) was discussed as indicator of iron availability but studies showed that sTfR values are altered during inflammation, too [15]. Consequently, the evaluation of iron homeostasis in patients suffering from inflammatory diseases is compromised. Moreover, the functional associations of alterations in markers of iron metabolism and the prognosis of septic patients are still incompletely understood. Several reports have shown that parameters of iron metabolism are predictors for outcome in ICU patients [16–19]. Nonetheless, there is still the need for more reliable biomarkers to predict the survival in ICU patients and to combine these with established clinical scores.

In the current report, we aimed to investigate the efficacy of serum iron parameters for predicting sepsis mortality in a prospective cohort of sepsis patients treated in an ICU. We identified a model including TF-Sat in sepsis prognostic scores like SOFA or SAPS II as a practically applicable and highly predictive model to improve the accuracy to assess the mortality risk in sepsis.

## Methods

### Study design and setting

This is a single-center prospective cohort study conducted at the medical intensive and intermediate care unit at the Medical University Innsbruck, Austria. Data were retrieved from consecutive patients admitted between February 2018 and December 2019. Patients were recruited prospectively, and iron parameters were analyzed retrospectively.

The study was carried out in accordance with the recommendations of the Declaration of Helsinki on biomedical research. The study was approved by the institutional ethics review board of the Medical University Innsbruck (study AN2013-0006). Parameters of iron metabolism were analyzed within 24h after study inclusion. Patients’ outcome was monitored for 28 days or until discharge from ICU. The manuscript was prepared in alignment to the *Strengthening the Reporting of Observational studies in Epidemiology* (STROBE) statement [20].

### Participants

Patients were screened within 24 h after admission to the ICU. Patients who were older than 18 years, who were not admitted to the ICU with status post cardiac resuscitation within the last 14 days and who presented with at least one confirmed or strongly suspected focus of infection, who also showed an altered mental status, a drop in systolic blood pressure (i.e., systolic blood pressure ≤ 100 mmHg), or an increased respiratory rate (qSOFA ≥ 2) were further screened for signs of organ failure. The primary inclusion criterion in the study was sepsis as defined according to the Sepsis-3 criteria: patients with suspected or confirmed infection and an increase in SOFA of at least two points [21]. Patients were excluded if (i) the patient was pregnant or breast feeding, (ii) the patient refused study participation, (iii) a patients’ life expectancy was estimated to be less than 24 h upon admission, (iv) if the primary reason for admission was cardiogenic shock, or (v) if the patients’ medical history indicated a pre-existing diagnosis of sepsis ≥ 24 h during the same hospital admission. Participants gave written informed consent prior to study inclusion or post hoc. Patient samples were stored and analyzed in anonymized form to ensure data confidentiality.

### Variables and measurements

The following parameters were obtained within 24 h following enrolment: age, sex, medical history, concomitant medication (especially immune-suppressive medication), application of red blood cell (RBC) transfusion within the last 3 months prior enrolment, sequential organ failure assessment score (SOFA), and simplified acute physiology score (SAPS II). All laboratory parameters including serum iron, ferritin, transferrin, and TF-Sat were done within 24 h after admission at the Central Institute of Medical and Chemical Laboratory Diagnostics, University Hospitals of Innsbruck. Immunosuppressive therapy was defined as therapeutic application of hydrocortisone (≥ 20 mg/day), tacrolimus, mycophenolate, cyclosporine, and/or rapamycin within the last 3 months prior to ICU admission. RBC transfusion was defined as application of at least one unit of RBC during the last three months prior to ICU admission. Documented clinical data included body temperature, blood pressure, heart rate, respiratory rate and use of vasopressors. Additionally, citrated blood samples were collected from an arterial line and platelet-free plasma was prepared as previously reported [22]. Platelet-free plasma samples were stored at – 80 °C. The main outcome variable was ICU mortality.

### Statistics

Statistical analysis was performed with R programming suite and tidyverse package bundle for data transformation. Continuous variables are tested for normal distribution with Shapiro-Wilk’s test and presented as mean and standard deviation or median and interquartile ranges (25th–75th percentile), respectively. Statistical significance for two-variable correlations and two-group differences were analyzed with ordinal linear regression and two-tailed *t* tests (Additional file 3 and Additional file 8), one-way ANOVA (Additional file 2), and mixed-effect linear modeling (package lme4 and lmerTest; fixed effect: variable of interest, random effects: age stratified by bi-decades and gender; Fig. 2 and Additional file 4). Correlation of the given variables with ICU mortality was assessed with logistic regression with inclusion of age and gender confounders (generalized linear model, logit transformation of the response, Wald *Z* tests for estimate significance; Fig. 3, Additional files 5, 9, and 10). Significance in predictive power of two nested models was determined by likelihood ratio test (LRT). Receiver-Operator Curve analysis and visualization was performed with the OptimalCutpoints and plotROC R packages, optimal cutoffs for the analyzed models were determined with Youden’s method (Fig. 4, Additional files 6 and 7). Test *p* values were corrected for multiple comparisons with Benjamini-Hochberg method.

Plotting was accomplished with ggplot2 package. Correlation results are presented as dot plots, where each point denotes single observation, blue lines represent fitted linear trends, and gray regions depict 95% confidence regions. Two-group comparison results are presented as bar plots (mean with SEM) with dot overlay, where each point represents a single observation. Survival modeling results are presented as forest plots, where points represent exponentiated model estimates, whiskers represent 95% CI, and labels displaying exponentiated estimate, CI, and *p* values.

## Results

### Characteristics of the study population

**Fig. 1:**
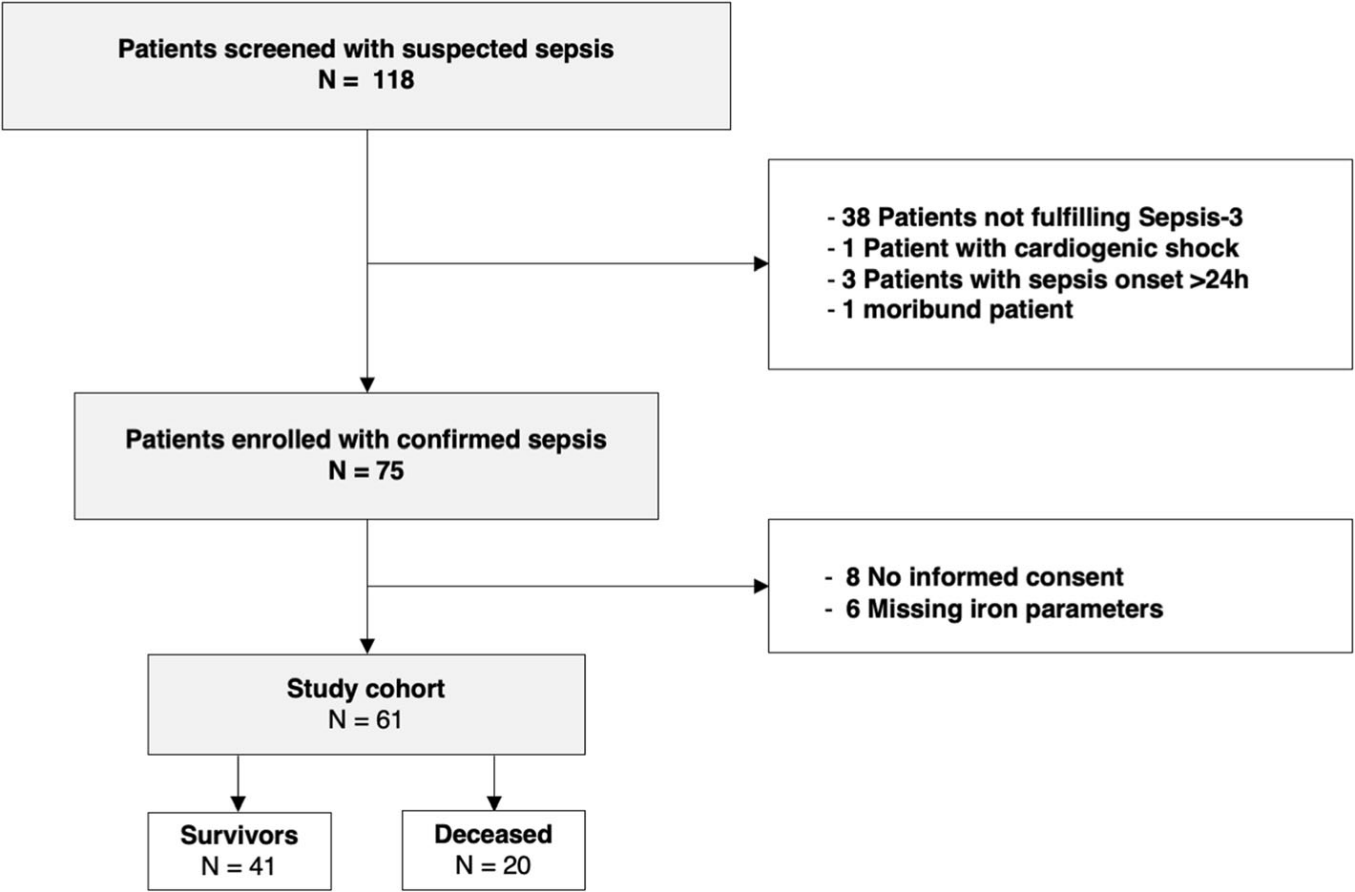
Flow chart of screening and inclusion of patients admitted to the ICU

During the study period, 118 patients admitted to the ICU at the Medical University of Innsbruck were screened and finally 61 patients with sepsis were enrolled (Fig. 1) of whom 47 (77.1%) had septic shock. The age of the included patients was 63 years on average, 60.7% were male. Upon admission, the mean SOFA score was 11 (± 4), and the mean SAPS II score 54 (± 19). Pneumonia was identified as the most common primary source of infection in 37.7% of cases, followed by urinary tract infections (12.8%). ICU mortality was 31.2%. Hematologic malignancies were present in 18%. The median white blood cell count was 13.6 G/l (6.5–20.8 G/l), the average concentration of CRP was 25 mg/dl (± 12 mg/dl). Serum iron parameters were severely altered in septic patients being compatible with inflammation mediated alterations of iron homeostasis [4] (Additional file 1). Decreased levels of serum iron (median 3.9 μmol/l, 2.3–7.4 μmol/l), of transferrin (mean 127.9 mg/dl ± 54.2) along with a reduced TF-Sat (median 11%, 7–33%) were observed whereas ferritin levels were above normal (median 567.5 μg/l, 254.5–1381 μg/l). Detailed patient characteristics are presented in Table 1. Laboratory parameters at admission are shown in Table 2.

**Table 1:** Characteristic of patients

	All patients (*n* = 61)	Survivor (*n* = 41)	Non-survivor (*n* = 20)	*p* value
Male, *n*	37 (60.7%)	22 (53.7%)	15 (75%)	0.163
Female, *n*	24 (39.3%)	19 (46.3%)	5 (25%)	0.163
Age, years	63 (21–88)	63 (21–88)	67 (53-85)	0.293
SOFA	11 (± 4)	10 (± 3.8)	14 (± 3.2)	0.0002
SAPS II	54 (± 19)	47 (± 16.6)	68 (± 16.9)	0.002
ICU-mortality, *n*	19 (31.2%)			
Hospital mortality, *n*	15 (23.4%)			
Pneumonia, *n*	23 (37.7%)	15 (36.6%)	8 (40%)	0.990
Urinary tract infection, *n*	6 (12.8%)	5 (12.2%)	1 (5%)	0.654
Acute abdominal infection, *n*	6 (9.8%)	3 (7.3%)	3 (15%)	0.384
Skin or soft tissue infection, *n*	6 (9.8%)	4 (9.8%)	2 (10%)	0.990
Blood catheter infection, *n*	2 (4.3%)	2 (4.8%)	0	0.990
Implant infection, *n*	1 (2.1%)	1 (2.4%)	0	0.990
Others, *n*	6 (9.8%)	3 (7.3%)	3 (15%)	0.384
Multiple foci, *n*	11 (18.0%)	6 (14.6%)	5 (25%)	0.479
patients with malignant diseases, *n*	11 (18.0%)	6 (14.6%)	5 (25%)	0.479
Septic shock, *n*	47 (77.1%)	28 (68.3%)	19 (95%)	0.024
Immunosuppresive co-medication, *n*	26 (41.0%)	15 (36.6%)	11 (55%)	0.270
Red blood cell transfusion (within 3 months before admission, *n*)	18 (29.5%)	7 (17.0%)	11 (55%)	0.006

Indicated are numbers of patients and in brackets the respective percentage to the cohort (all patients, survivor, non-survivor)

Age is reported as median and range, SOFA and SAPS II scores are reported as mean ± SD

*p* values were determined by Fisher’s exact test, *t* test, Mann-Whitney test

**Table 2:** Laboratory parameters at admission

	All patients (*n* = 61)	Survivor (*n* = 20)	Non-survivor (*n* = 41)	*p* value
WBC (G/l)	13.6 (6.5–20.8)	14.6 (7.4–21)	12.4 (2.2–19.4)	0.6558
C-reactive protein (mg/dl)	25.0 (± 12)	24.3 (± 11.2)	26.3 (± 13.2)	0.550
Creatinine (mg/dl)	2.1 (1.3–3.2)	1.8 (1.3–2.9)	2.2 (0.9–2.7)	0.930
Iron (μmol/l)	3.9 (2.3–7.4)	3.3 (2.2–6.2)	6.1 (2.9–12.5)	0.041
Ferritin (μg/l)	567.5 (254.5–1381)	395 (203.5–834.5)	1558 (322–3967)	0.008
Transferrin (mg/dl)	127.9 (± 54.2)	138.4 (± 51.3)	106.3 (± 52.2)	0.034
TF-Sat (%)	11 (7–33.5)	9 (6–19)	25 (8–59.5)	0.021

Parameters with normal distribution are presented as mean ± SD, measurements with significant deviation from a normal distribution (Shapiro-Wilk *p* < 0.05) are presented as median and interquartile range. *WBC* white blood cell count, *TF-Sat* transferrin saturation

### Iron parameters and mortality

**Fig. 2:**
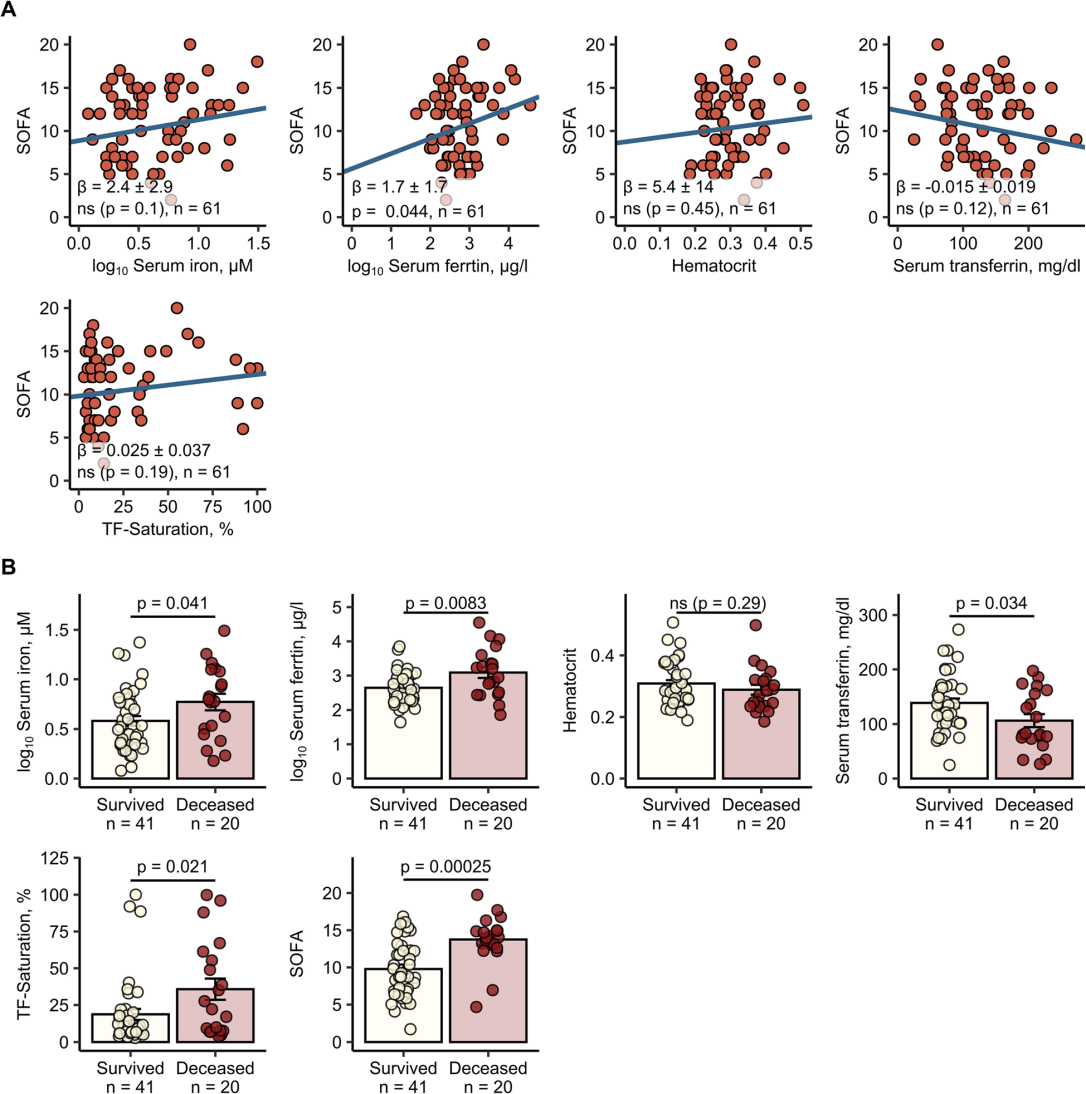
Iron parameters correlate with SOFA and vital status of sepsis patients. Iron parameters and hematological markers: serum iron, ferritin, hematocrit, serum transferrin, and TF-Sat and SOFA score were routinely determined at patient’s admission at ICU. **a** Correlation of iron parameters with SOFA. SOFA score value was modeled as a function of the parameters with mixed-effect linear model (fixed effect: the investigated parameter, random effects: gender and age). For strongly non-normally distributed ferritin and iron concentrations log_10_ values were used for modeling. Each point represents a single observation, and blue solid lines depict the fitted regression lines. Regression slope β estimates with 95% CI, *p* value for slope significance (two-tailed *t* test, β ≠ 0) and n numbers of observations are presented in the plots. **b** Differential regulation of iron parameters in survivors and ICU deceased sepsis patients. Each point represents a single observation, bars and whiskers depict mean with SEM. Statistical significance was determined with mixed-effect linear model (fixed effect: vital status, random effects: gender and age). For strongly non-normally distributed ferritin and iron concentrations log_10_ values were used for modeling. In the plots, n numbers of survivors and deceased participants and p values (two-tailed *t* test, β ≠ 0) are presented

No differences in iron parameter distribution were observed when patients were stratified according to the source of infection (Additional file 2) or when the group suffering from pneumonia was compared with other infections (Additional file 3). However, SOFA score in patients with urinary tract infections was significantly lower compared to subjects with other infectious foci (Additional file 2). Serum ferritin levels significantly correlated with SOFA score at admission (*p* = 0.044) (Fig. 2a) and significant positive associations of serum iron and ferritin levels with another prognostic score, SAPS II were identified (Additional file 4AB). Survivors had significant lower serum iron (*p* = 0.041), lower serum ferritin (*p* = 0.0083), lower TF-Sat (*p* = 0.021), and higher serum transferrin (*p* = 0.034) as compared with the non-survivor subset (Fig. 2b, Table 2).

**Fig. 3:**
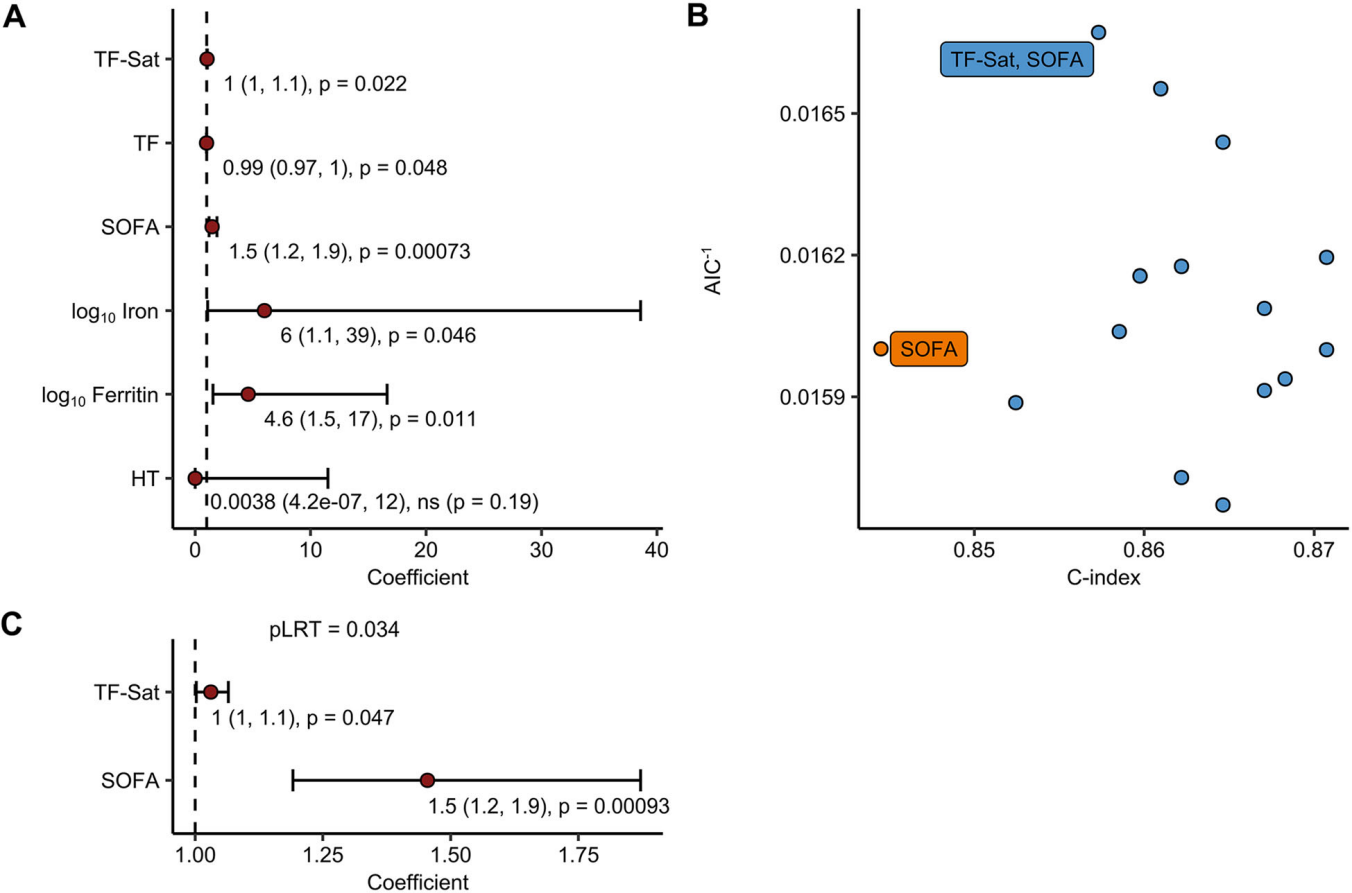
Transferrin saturation is a SOFA-independent survival predictor. **a** Predictive power of age- and sex-corrected serum iron concentration, ferritin, transferrin (TF), TF saturation (TF-Sat), hematocrit (HT), and SOFA. Correlation with ICU mortality for each of the investigated parameters was analyzed with a separate logistic regression model with inclusion of age and sex as confounders. Significance for regression β estimates was determined with Wald *Z* test. Results are presented as a forest plot. Points represent exponentiated β estimates, and whiskers represent 95% CI. Points are labeled with exponentiated estimate, CI, and *p* values. Survivors: *n* = 41, deceased: *n* = 20. **b** Comparison of predictive power for SOFA and compound SOFA-iron parameter models. A family of logistic regression models correlating SOFA, 0–3 combinations of the iron/hematological parameters linked in **a** to survival as well as of age and sex as confounders with ICU mortality was generated. For each model, estimate p-values (determined with Wald *Z* test), Akaike Information Criterion (AIC) and concordance index (C-index) was calculated. Best performing models, i.e., those with significant non-confounder estimates and better AIC and C-index than the SOFA-alone model, are labeled with non-confounder variable names. Survivors: *n* = 41, deceased: *n* = 20. **c** Estimates for non-confounder parameters of the best-performing SOFA-compound model identified in (B, SOFA + TF-Sat) presented as a forest plot. Significance for regression β estimates was determined with Wald *Z* test. Points represent exponentiated β estimates, whiskers represent 95% CI. Points are labeled with exponentiated estimate, CI, and *p* values. Significance of the inclusion of the TF-Sat term was assessed with likelihood ratio test (*p*_LRT_, compound model vs SOFA-alone model). Survivors: *n* = 41, deceased: *n* = 20

Based on this observation, we questioned whether specific iron parameters alone or in combination with other demographic parameters or risk factors including the initial SOFA (*p* = 0.00025 between ICU survivors and non-survivors, Fig. 2b) and SAPS II score (*p* = 0.00019 between survivors and non-survivors, Additional file 4A) predict outcome in septic patients. Initially, correlations with ICU mortality for each of the investigated parameters were analyzed with a separate logistic regression model with inclusion of age and sex as confounders. SOFA and SAPS II as well as all iron parameters measured upon ICU admission, except for hematocrit, significantly correlated with a higher risk of ICU death (Fig. 3a, Additional file 5A, Table 3; TF-Sat *p* = 0.022, TF *p* = 0.048, SOFA *p* = 0.00073, serum iron *p* = 0.046, serum ferritin *p* = 0.011, hematocrit *p* = 0.19, SAPS II *p* = 0.00064). Next, independence of the SOFA and the studied iron parameters as markers of mortality risk was tested. Therefore, a family of SOFA-extended models consisting of SOFA score and 1–3 combinations of iron parameters was created and their predictive potential for ICU mortality was studied with logistic regression including age and sex as confounders. As presented in Fig. 3b and Table 3, each of these models demonstrated higher predictive power than SOFA-alone as demonstrated by higher concordance index (C-index). In addition, 8 models, including SOFA, transferrin, TF-Sat, iron, and combinations thereof, demonstrated a better data fit than SOFA alone as indicated by lower AIC (Akaike’s Information Criterion) values. Two models, SOFA with serum ferritin (*p* = 0.043) and SOFA with TF-Sat (*p* = 0.034), showed also a superior predictive power as compared to the use of SOFA alone in the LRT test (Table 3). In those two models, only TF-Sat retained its significant positive correlation with mortality despite SOFA inclusion (*p* = 0.047, Wald *Z* test), suggesting that TF-Sat can be regarded as a SOFA independent predictor of mortality (Fig. 3c). Cumulatively, these results suggest that the compound SOFA–TF-Sat model may differentiate better between sepsis survivors and non-survivors than SOFA-alone. We next sought to check if TF-Sat may also act as an SAPS II independent prognostic factor. Inclusion of TF-Sat substantially improved the accuracy of SAPS II at predicting ICU mortality and model fit in terms of C-index, AIC (Additional file 5B, Table 3) and better performance in LRT test (Additional file 5C, Table 3). However, the TF-Sat term did not retained statistical significance after inclusion of SAPS II in the model (*p* = 0.060, Wald *Z* test) and, hence, could not be considered a SAPS II independent prognostic marker (Additional file 5C).

**Table 3:** Characteristics of SOFA and SOFA-extended logistic regression models presented in Fig. 3b

Model ID	Parameters	P LRT	AIC	C-index
SOFA_1	log10 ferritin, SOFA, sex, age	0,043	60	0.86
SOFA_2	log10 iron, SOFA, sex, age	0,21	63	0.85
SOFA_3	Transferrin, SOFA, sex, age	0,13	62	0.87
SOFA_4	TransfSat, SOFA, sex, age	0,034	60	0.86
SOFA_5	log10 ferritin, log10 iron, SOFA, sex, age	0,13	62	0.86
SOFA_6	log10 ferritin, transferrin, SOFA, sex, age	0,093	62	0.87
SOFA_7	log10 ferritin, TF-Sat, SOFA, sex, age	0,059	61	0.86
SOFA_8	log10 iron, transferrin, SOFA, sex, age	0,16	63	0.87
SOFA_9	log10 iron, TF-Sat, SOFA, sex, age	0,1	62	0.86
SOFA_10	Transferrin, TF-Sat, SOFA, sex, age	0,097	62	0.86
SOFA_11	log10 ferritin, log10 iron, transferrin, SOFA, sex, age	0,18	64	0.86
SOFA_12	log10 ferritin, log10 iron, TF-Sat, SOFA, sex, age	0,11	63	0.87
SOFA_13	log10 ferritin, transferrin, TF-Sat, SOFA, sex, age	0,12	63	0.87
SOFA_14	log10 iron, transferrin, TF-Sat, SOFA, sex, age	0,2	64	0.86
SOFA	SOFA, sex, age	NA	62	0.84
SAPSII_1	log10 ferritin, SAPSII, sex, age	0,041	61	0.88
SAPSII_2	log10 iron, SAPSII, sex, age	0,28	64	0.86
SAPSII_3	Transferrin, SAPSII, sex, age	0,091	62	0.87
SAPSII_4	TransfSat, SAPSII, sex, age	0,049	61	0.87
SAPSII_5	log10 ferritin, log10 iron, SAPSII, sex, age	0,12	63	0.88
SAPSII_6	log10 ferritin, transferrin, SAPSII, sex, age	0,071	62	0.88
SAPSII_7	log10 ferritin, TF-Sat, SAPSII, sex, age	0,068	62	0.88
SAPSII_8	log10 iron, transferrin, SAPSII, sex, age	0,16	63	0.87
SAPSII_9	log10 iron, TF-Sat, SAPSII, sex, age	0,13	63	0.87
SAPSII_10	Transferrin, TF-Sat, SAPSII, sex, age	0,11	63	0.87
SAPSII_11	log10 ferritin, log10 iron, transferrin, SAPSII, sex, age	0,15	64	0.88
SAPSII_12	log10 ferritin, log10 iron, TF-Sat, SAPSII, sex, age	0,11	63	0.88
SAPSII_13	log10 ferritin, transferrin, TF-Sat, SAPSII, sex, age	0,13	63	0.88
SAPSII_14	log10 iron, transferrin, TF-Sat, SAPSII, sex, age	0,22	65	0.88
SAPSII	SAPSII, sex, age	NA	63	0.85

*FT* ferritin, *TF* transferrin, *TF-Sat* transferrin saturation, *Iron* serum iron

**Fig. 4:**
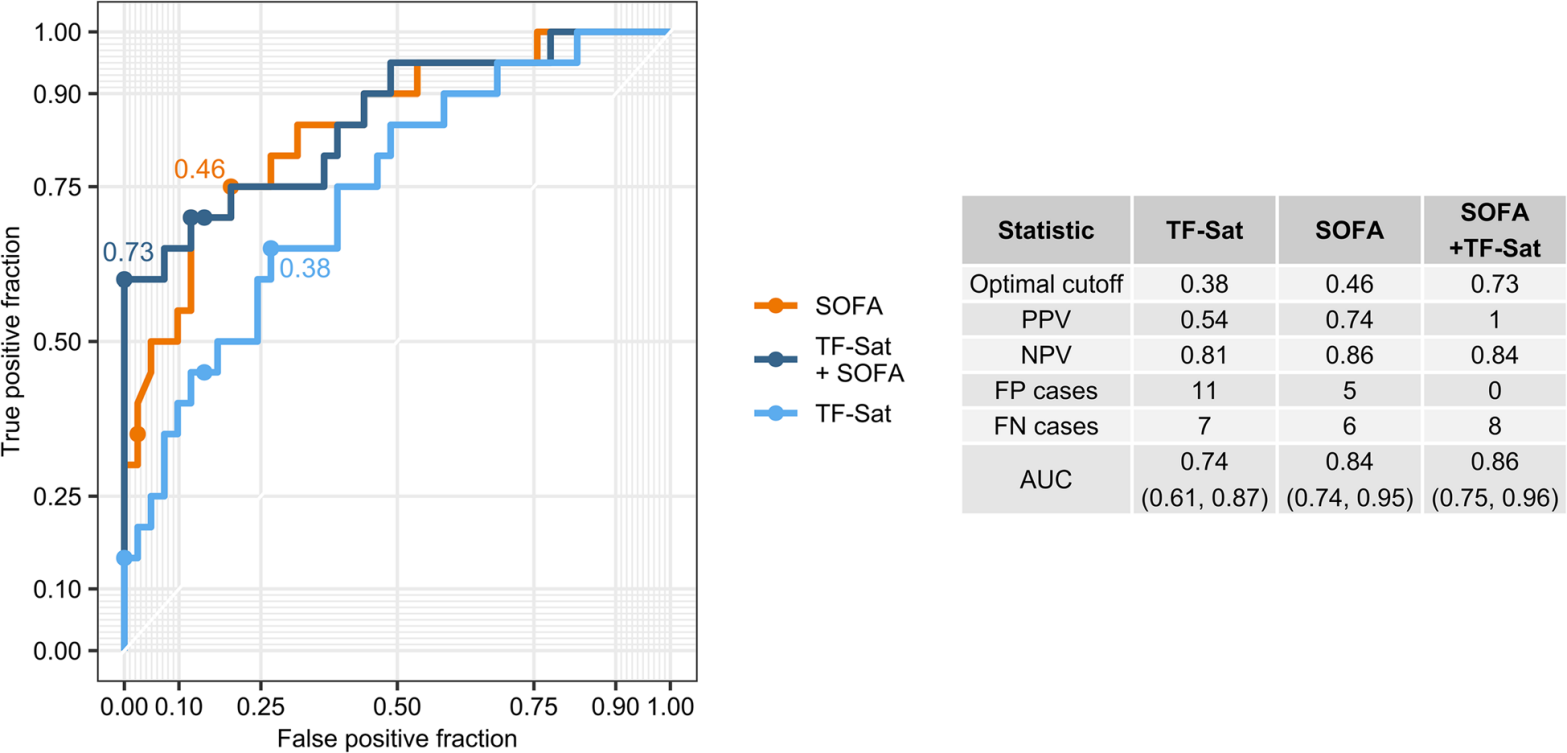
Predictive power of transferrin saturation in receiver-operator curve (ROC) analysis. Theoretical survival probabilities for study participants were calculated using the age- and sex-adjusted SOFA, age- and sex-adjusted TF-Sat and the age- and sex-adjusted SOFA-compound model identified in Fig. 3b (SOFA + TF-Sat). The plot displays ROC for the models with optimal cutoffs labeled. The table presents calculated optimal cutoffs, positive and negative predictive values at the optimal cutoff (PPV and NPV), numbers of false-positive and false-negative cases at the optimal cutoff (FP and FN), and area under the curve (AUC) for ROCs with 95% CI

Finally, we compared the specificity and sensitivity of TF-Sat-alone, SOFA-alone, SAPS II-alone as well as the SOFA-TF-Sat and SAPS II-TF-Sat compound scores at predicting ICU mortality in a receiver-operator curve (ROC) analysis. TF-Sat alone demonstrated significant predictive power (AUC 0.74, 95% CI 0.61–0.87), but less than the SOFA score (AUC 0.84, 95% CI 0.74–0.95). The compound SOFA-TF-Sat score showed only slightly improved prediction accuracy (AUC 0.86, 95% CI 0.75–0.96) as compared with the SOFA-alone model (Fig. 4). However, the inclusion of TF-Sat substantially improved the overall (Additional file 7A, 9 false predictions in the compound model versus 12 false predictions in the SOFA-alone model, 61 predictions in total) and positive prediction power of the SOFA-alone model, resulting in elimination of false-positive cases (Fig. 4). Importantly, an analogical ROC analysis performed with the SAPS II-alone and the SAPS II–TF-Sat models revealed fairly similar tendencies including the improved positive (4 false-positive cases identified by the SAPS II–TF-Sat models versus 9 identified by the SAPS II-alone model, Additional File 6) and overall prediction values (10 false predictions for the SAPS II–TF-Sat model versus 13 false predictions for the SAPS II-alone model, 61 predictions in total**,** Additional File 7B). Of interest, although both the SOFA- and SAPS II-alone models correctly identified all non-survivors among the patients demonstrating TF-Sat above the normal level of 45%, inclusion of the TF-Sat parameter in the models substantially improved the accuracy of prediction in the subset with TF-Sat levels below 45% (9 false predictions for SOFA–TF-Sat versus 12 false predictions for SOFA-alone model, 51 predictions total, Additional figure 7B). In particular, the subjects with high values of SAPS II (above 50) corresponding to over 40% estimated mortality [23, 24] and normal to low TF-Sat values tend to have higher survival chances than inferred from the SAPS II score-alone (8 false predictions for SAPS II–TF-sat versus 11 false predictions for SAPS II-alone model, 33 predictions in total, Additional file 7B). Cumulatively, we put forward TF-Sat as a both standalone predictor of sepsis survival and as an iron parameter which may improve the prediction power of SOFA an SAPS II by reduction of the rate of false-positive cases.

### Influence of red-blood cell transfusions and immunosuppressive co-medication on the predictive power of the SOFA–TF-Sat-compound model

Several of the study participants received RBC transfusions (*n* = 18, 29.5%) or were on immunosuppressive co-medication (*n* = 26, 41.0%) in the last 3 months before ICU admission. We asked hence, if these two factors impacted on the levels of iron parameters and their correlation with mortality, with and without inclusion of SOFA. RBC transfusions may be regarded as a form of iron supplementation. One human RBC unit contains 220 to 250 mg of iron [25] and high circulating iron or heme levels may promote intravascular oxidative stress and inflammation but also serve as a source for pathogens [26]. In our cohort, individuals having received RBC transfusions within the last 3 months before ICU admission displayed higher serum iron, ferritin and TF-Sat values and significantly lower hematocrit levels as compared to non-transfused sepsis patients at initial presentation to the ICU. Of note, previous administration of RBC transfusions was not linked to higher SOFA scores (Additional file 8A). Results of logistic regression modeling of ICU mortality in our collective indicate that RBC transfusion (*p* = 0.0067, Wald *Z* test) but not immunosuppression was associated with a higher death risk when analyzed with separate models corrected for the effects of age and sex (Additional file 9A). Inclusion of the SOFA score in such models shows that RBC transfusion (pLRT = 0.0028 vs SOFA-alone model, LRT test; pRBC = 0.0073 for the RBC transfusion term, Wald *Z* test, Additional file 9B) but not immunosuppressive therapy (pLRT = 0.53 vs SOFA-alone model, LRT; pImmunosuppression = 0.53 for the immunosuppression term, Wald *Z* test, Additional file 9C), can be regarded as a SOFA-independent prognostic factor.

Next, we investigated whether RBC transfusions or immunosuppression affected the prognostic power of the best-performing SOFA–TF-Sat-compound model. While inclusion of immunosuppressive co-medication (*P*_LRT_ = 0.85) did not alter the predictive power of SOFA–TF-Sat, inclusion of RBC transfusion into the SOFA–TF-Sat model basically neutralized the effect of TF-Sat on mortality (*P*_LRT_ = 0.017, pTF-Sat = 0.27 for the TF-Sat term, Wald *Z* test) (Additional file 10). Cumulatively, this indicates that TF-Sat is a SOFA independent but RBC transfusion dependent prognostic factor for ICU death risk.

## Discussion

This study aimed to investigate the efficacy of serum iron parameters for predicting sepsis mortality in a prospective cohort of sepsis patients treated in an ICU and identified TF-Sat as additional laboratory parameter to increase the predictive power of the SOFA score, yielding our SOFA - TF-Sat model.

The importance of iron metabolism on all-cause mortality in ICU patients and specifically septic patients has been previously described. Alterations of iron homeostasis in the setting of sepsis are driven by cytokines and acute phase proteins along with their effects on erythropoiesis and erythrocyte damage. While these subtle alterations are considered as a defense strategy of the body against invading microbes, alterations of iron homeostasis may negatively impact on metabolic processes of cells including mitochondrial respiration, immune function, or tissue oxygen supply [27, 28].

Accordingly, patients in our cohort presented with alterations of iron homeostasis parameters being typical for inflammation and infection driven alterations, namely decreased serum iron levels and TF-Sat, decreased serum transferrin levels, and an increase of ferritin concentrations [3, 29].

Transferrin is a negative acute phase protein because IL-6 inhibits its production by the liver parenchyma [30]. Although transferrin binds iron with high affinity, transferrin-bound iron remains potentially accessible for microbes. Many microbes including Gram-negative Enterobacteriaceae produce siderophores whose affinity for iron exceed that of transferrin by several orders of magnitude [31]. Therefore, these microbes may be able to strip iron off transferrin and use it for their own growth and proliferation. Elevated serum ferritin is an indicator of both iron storage in tissues and inflammation. All nucleated cell types express ferritin in order to store iron [14]. Within cells, ferritin is essential to avoid iron-induced formation of reactive oxygen species and thus cellular toxicity and to limit the access to iron for microbes [32]. Macrophages display a high capacity to either store iron in ferritin or secrete ferritin by non-classical pathways and are therefore considered the major source of serum ferritin [33]. It is thus possible that the high serum ferritin levels observed in non-survivors are a sign of increased macrophage activation rather than increased iron stores [34].

The influence of RBC transfusion on the predictive power of the TF-Sat–SOFA-compound model and the influence of RBC transfusion on the survival of septic patients in our study is in accordance with reports of increased rates of infection and sepsis in post-operative patients receiving RBC transfusions [35], increased risk of sepsis in pediatric burn patients [36], and increased risk of ICU-acquired infections in the critically ill [37].

Substantial amounts of free heme and iron are set free during the lysis of RBC before and during transfusions. Furthermore, non-transferrin bound iron may transiently appear after RBC transfusion. Therefore, various forms of iron may exert toxic effects because of their prooxidative properties, and they may impair host immunity or serve as iron source for microbes [26, 38]. However, in a multicentric observation study including more than 3000 patients with sepsis, no clear association between RBC transfusions and mortality was observed [39]. In line with in our single-center prospective study, the previous administration of RBC transfusions was not linked to higher SOFA scores. In contrast, we found an association of high TF-Sat and low transferrin concentrations with reduced survival. This could be due to the fact that some patients may be able to handle the iron contained in packed RBCs as their macrophages can store it intracellularly or release it continuously thus meeting the demand of the erythron. In other patients, however, the storage or regulated recycling capacity of macrophages may be overwhelmed so that peak amounts of exported iron result in excessive TF-Sat. Therefore, an elevated TF-Sat may indicate a dysfunction of the macrophage system during sepsis. This idea is in line with other studies demonstrating that high serum iron and TF-Sat [14, 16] as well as serum ferritin [15] were associated with mortality from sepsis. On the other hand, iron deficiency as diagnosed from low hepcidin levels, predicted 1-year mortality after ICU discharge [17]. There are several possible explanations for the discrepancies between these studies. First, the combination of high serum iron, TF-Sat and ferritin may primarily be attributable to immune-driven alterations of iron metabolism whereas suppressed hepcidin is a clear although not well-standardized indicator of absolute iron deficiency. Second, patient outcome is highly dependent on the underlying disease and the subgroup of patients with sepsis may be underrepresented in studies that also investigated ICU patients without sepsis [17]. Third, the design of studies investigating iron parameters in the critically ill is variable.

The strengths of our study are its prospective design and its focus on patients with sepsis. Although our sample size is limited, we identified TF-Sat, determined within 24 h of admission to intensive care, as additional laboratory parameter to increase the predictive power of the SOFA score. Both values are routinely assessed in sepsis patients, so no additional clinical parameters would be needed. Statistical analysis proved TF-Sat as an independent value of SOFA and as a nearly independent value of SAPS II. Two goodness measures showed that the combination of the SOFA score with TF-Sat better identifies survivors and non-survivors than the SOFA score alone. Furthermore, combining the SOFA score with TF-Sat had a significantly lower residual variance number when the likelihood ratio test was applied meaning that the number of unexplained ICU deaths and survivors is expected to be lower when TF-Sat is included in addition to SOFA. In addition, ROC analysis showed that the compound model substantially reduced the number of false predictions and virtually eliminated false-positive cases at the optimal cutoff. All together, the inclusion of TF-Sat in the widely used sepsis prognostic scores like SOFA and, to a lesser extent, SAPS II may improve their accuracy and enhance predictions especially for patients demonstrating high score values but low TF-Sat.

However, our study has several limitations. First, it was conducted as a single-center study and limited to patients treated in a medical ICU. Second, there were only few patients with abdominal or urinary tract infections which have been found to be associated with lower in hospital-mortality [40]. However, the distribution of sepsis-foci is in line with other studies, and serum iron parameters were not associated with the site of infection confirming that our findings are representative. Third, the sample size of our study cohort was limited.

Nevertheless, the present study provides a combination model of SOFA + TF-Sat as a practically applicable and highly predictive model to assess the mortality risk in sepsis. This supports the finding of novel associations of iron parameters and disease outcome which may be of importance for clinical management of patients at risk.

Yet, it will be important to validate these findings in larger prospective multicenter studies.

## Conclusion

Positive correlations of increased serum iron and ferritin concentrations upon ICU admission with the SOFA score and with mortality were observed. Moreover, high TF-Sat and low-transferrin concentrations were associated with reduced survival. A logistic regression model consisting of SOFA and TF-Sat had the best predictive power for survival in septic ICU patients. The study discloses previously underestimated interactions between systemic iron homeostasis and the clinical course of sepsis which may be linked to iron mediated effects on microbial growth, immune function, and/or radical formation.

## Supplementary information

**Additional file 1.** Overview of systemic iron turnover at steady state and during infection or inflammation. At steady-state (upper panel) levels of hepcidin, the inhibitor of ferroportin-1 (FPN1) – mediated cellular iron export, are low. This enables efficient dietary iron uptake in the duodenum and recycling of iron derived from aged and damaged red blood cells (RBC) in macrophages. As a result, iron saturation of circulating transferrin (TF) stays at physiological levels and sufficient iron is available for bone marrow erythropoiesis and other body organs. The excess iron is stored in the liver and spleen. During infection or inflammation (lower panel), hepcidin levels rise, hence blocking the FPN1-mediated dietary iron uptake and iron recycling in macrophages. Circulating iron is, in addition, extensively captured and stored by macrophages, the liver and spleen. Cumulatively, TF saturation decreases and iron availability for erythropoiesis, other cells of the body but also for potential pathogens such as bacteria, viruses or fungi is very low. If chronic, inflammation or infection may lead to reduced RBC hemoglobinization and cellularity manifested as anemia of chronic disease (ACD).

**Additional file 2.** Regulation of iron turnover markers in study participants stratified by the primary infection foci. Each point represents a single observation, bars and whiskers depict mean with SEM. Statistical significance was determined with one-way ANOVA and Benjamini-Hochberg post-hoc two-tailed T tests. Strongly non-normally distributed ferritin and iron concentrations were log_10_ transformed. In the plots, n numbers of patients in each focus strata and significant post-hoc test p values are presented. ANOVA statistics: serum iron: F_5, 55_ = 0.81, ns; serum ferritin: F_5, 55_ = 0.12, ns; hematocrit: F_5, 55_ = 0.46, ns; transferrin: F_5, 55_ = 0.31, ns; transferrin saturation (TF-Sat): F_5, 55_ = 0.12, ns; SOFA: F_5, 55_ = 3.0, p = 0.018.

**Additional file 3.** Regulation of iron turnover markers in study participant suffering from pneumonia and other infections. Regulation of iron turnover markers and SOFA in pneumonia patients and participants suffering from other infections. Each point represents a single observation, bars and whiskers depict mean with SEM. Statistical significance was determined with two-tailed T-test. Strongly non-normally distributed ferritin and iron concentrations were log_10_ transformed. In the plots, n numbers of pneumonia and other infection individuals and p values (two-tailed T test) are presented.

**Additional file 4.** Correlation of iron turnover parameters with SAPS II. (A) Values of SAPS II in survivors and ICU deceased sepsis patients. Each point represents a single observation, bars and whiskers depict mean with SEM. Statistical significance was determined with mixed-effect linear model (fixed effect: vital status, random effects: gender and age). In the plot, n numbers of survivors and deceased participants and p value (two-tailed T test, β ≠ 0) are presented. (B) Correlation of iron parameters with SAPS II. SAPS II score value was modeled as a function of the iron turnover parameters with mixed-effect linear model (fixed effect: the investigated parameter, random effects: gender and age). For strongly non-normally distributed ferritin and iron concentrations log_10_ values were used for modeling. Each point represents a single observation, blue solid lines depict the fitted regression lines. Regression slope β estimates with 95% CI, p value for slope significance (two-tailed T test, β ≠ 0) and n numbers of observations are presented in the plots.

**Additional file 5.** Dependence of transferrin saturation on SAPS II in predicting ICU survival. (A) Predictive power of age- and sex-corrected serum iron concentration, ferritin, transferrin (TF), TF saturation (TF-Sat), hematocrit (HT) and SAPS II. Correlation with ICU mortality for each of the investigated parameters was analyzed with a separate logistic regression model with inclusion of age and sex as confounders. Significance for regression β estimates was determined with Wald Z test. Results are presented as a forest plot. Points represent exponentiated β estimates, whiskers represent 95% CI. Points are labeled with exponentiated estimate, CI and p values. Survivors: n = 41, deceased: n = 20. (B) Comparison of predictive power for SAPS II and compound SAPS II-iron parameter models. A family of logistic regression models correlating SAPS II, 0 - 3 combinations of the iron/hematological parameters linked in (A) to survival as well as of age and sex as confounders with ICU mortality was generated. For each model, estimate p-values (determined with Wald Z test), Akaike Information Criterion (AIC) and concordance index (C-index) was calculated. The SAPS II-alone model and the SAPS II - TF-Sat compound model are labeled in the plot. Survivors: n = 41, deceased: n = 20. (C) Estimates for the SAPS II - TF-Sat compound model presented as a forest plot. Significance for regression β estimates was determined with Wald Z test. Points represent exponentiated β estimates, whiskers represent 95% CI. Points are labeled with exponentiated estimate, CI and p values. Significance of the inclusion of the TF-Sat term was assessed with likelihood ratio test (p_LRT_, compound model vs SAPS II-alone model). Survivors: n = 41, deceased: n = 20.

**Additional file 6.** Predictive power of transferrin saturation in receiver-operator curve (ROC) analysis. Theoretical survival probabilities for study participants were calculated using the age- and sex-adjusted SAPS II, age- and sex-adjusted TF-Sat and the age- and sex-adjusted SAPS II compound model presented in Figure S5B (SAPS II - TF-Sat). The plot displays ROC for the models with optimal cutoffs labeled. The table presents calculated optimal cutoffs, positive and negative predictive values at the optimal cutoff (PPV and NPV), numbers of false-positive and false-negative cases at the optimal cutoff (FP and FN) and area under the curve (AUC) for ROCs with 95% CI.

**Additional file 7.** Accuracy of ICU mortality prediction by the SOFA - TF-Sat and SAPS II - TF-Sat compound models. Theoretical survival probabilities for study participants were calculated using (A) the age- and sex-adjusted SOFA, age- and sex-adjusted TF-Sat and the age- and sex-adjusted SOFA compound model presented in Figure S5B (SOFA + TF-Sat) and (B) the age- and sex-adjusted SAPS II, age- and sex-adjusted TF-Sat and the age- and sex-adjusted SAPS II compound model presented in Figure S5B (SAPS II + TF-Sat). For each model, optimal cutoffs were determined by ROC analysis as presented in Figure 3 and Figure S6 and patients were stratified into predicted survivors and predicted deceased and the predicted survival was compared with the actual ICU mortality (correct or false prediction). Point plots depict (A) TF-Sat and SOFA and (B) TF-Sat and SAPS II values, points fill codes for the actual vital status, point shape for quality of model prediction. Plots are tagged with the absolute numbers of correct and false survival predictions.

**Additional file 8.** Regulation of iron turnover parameters in patients having been treated with RBC transfusions and immunosuppression before ICU admission. Study participants were stratified into the group who had been treated with red blood cell (RBC) transfusions or immunosuppressive co-medication within the last 3 months before ICU admission. (A) Regulation of iron parameters in RBC transfused and transfusion-naive study participants. Each point represents a single observation, bars and whiskers depict mean with SEM. Statistical significance was determined with two-tailed T-test. Strongly non-normally distributed ferritin and iron concentrations were log_10_ transformed. In the plots, n numbers of transfused and non-transfused individuals and p values (two-tailed T test) are presented. (B) Regulation of iron parameters markers in immunosuppression-treated (Sup) and immunosuppression-naive (No Sup) study participants. Each point represents a single observation, bars and whiskers depict mean with SEM. Statistical significance was determined with two-tailed T-test. Strongly non-normally distributed ferritin and iron concentrations were log_10_ transformed. In the plots, n numbers of immunosuppression-treated and -naive individuals and p values (two-tailed T test) are presented.

**Additional file 9.** Predictive power and SOFA dependence of RBC transfusions and immunosuppression for ICU survival. Study participants were stratified into the group who had been treated with red blood cell (RBC) transfusions or immunosuppressive co-medication within the last 3 months before ICU admission. SOFA score was routinely determined at ICU admission. (A) Predictive power of age- and sex-corrected RBC transfusion, immunosuppressive therapy and SOFA. Correlation with ICU mortality for each of the investigated parameters was analyzed with a separate logistic regression model with inclusion of age and sex as confounders. Significance for regression β estimates was determined with Wald Z test. Results are presented as a forest plot. Points represent exponentiated β estimates, whiskers code for 95% CI. Points are labeled with exponentiated estimate, CI and p values. Survivors: n = 41, deceased: n = 20, RBC transfused n = 18, treated with immnosuppression: n = 26. (B, C) Predictive power of RBC transfusion- and immunosuppression-confounded SOFA score for ICU mortality. Correlations for SOFA and RBC transfusion status (B) as well as SOFA and immunosuppression (C) with ICU mortality were analyzed with separate logistic regression models with inclusion of age and sex as confounders. Significance for regression β estimates was determined with Wald Z test. Results are presented as a forest plot. Points represent exponentiated β estimates, whiskers represent 95% CI. Points are labeled with exponentiated estimate, CI and p values. Significance of the inclusion of the RBC transfusion and the immunosuppression term was assessed with likelihood ratio test (p_LRT_, SOFA - RBC transfusion vs SOFA and SOFA - immunosuppression vs SOFA). Survivors: n = 41, deceased: n = 20, RBC transfused n = 18, treated with immnosuppression: n = 26.

**Additional file 10.** Effects of inclusion of the RBC and immunosuppression effects on the predictive value of the compound SOFA model. (A, B) Effects of inclusion of the RBC transfusion term (A) and the immunosuppression term (B) on the predictive value of the compound SOFA model. The best performing SOFA - TF-Sat compound model identified in Figure 2B was appended with terms coding for RBC transfusion status (A) and immunosuppressive medication (B). Significance for regression β estimates was determined with Wald Z test. Results are presented as a forest plot. Points represent exponentiated β estimates, whiskers represent 95% CI. Points are labeled with exponentiated estimate, CI and p values. Significance of the inclusion of the RBC transfusion and the immunosuppression term was assessed with likelihood ratio test (p_LRT_, SOFA - TF-Sat + RBC transfusion vs SOFA - TF-Sat, SOFA - TF-Sat + immunosuppression vs SOFA + TF-Sat). Survivors: n = 41, deceased: n = 20, RBC transfused n = 18, treated with immunosuppression: n = 25.

## Data Availability

The datasets used and/or analyzed during the current study are available from the corresponding author on reasonable request.

## Abbreviations

SOFA: Sepsis-related organ failure assessment score
SAPS II: Simplified acute physiology score
TF-Sat: Transferrin saturation ()
RBC: Red blood cells
ICU: Intensive care unit
CRP: C-reactive protein
HT: Hematocrit
PPV: Positive predictive value (
NPV: Neative predictive value
FP: False-positive
FN: False-negative
AUC: Area under the curve
CI: Confidence interval
AIC: Akaike’s Information Criterion
ANOVA: Analysis of variance
