# Supplementary figures and images for: Linkage of alterations in systemic iron homeostasis to patients’ outcome in sepsis: a prospective study
Source: J Intensive Care. 2020 Oct 1;8:76. doi: 10.1186/s40560-020-00495-8 (PMC7528491; doi:10.1186/s40560-020-00495-8)

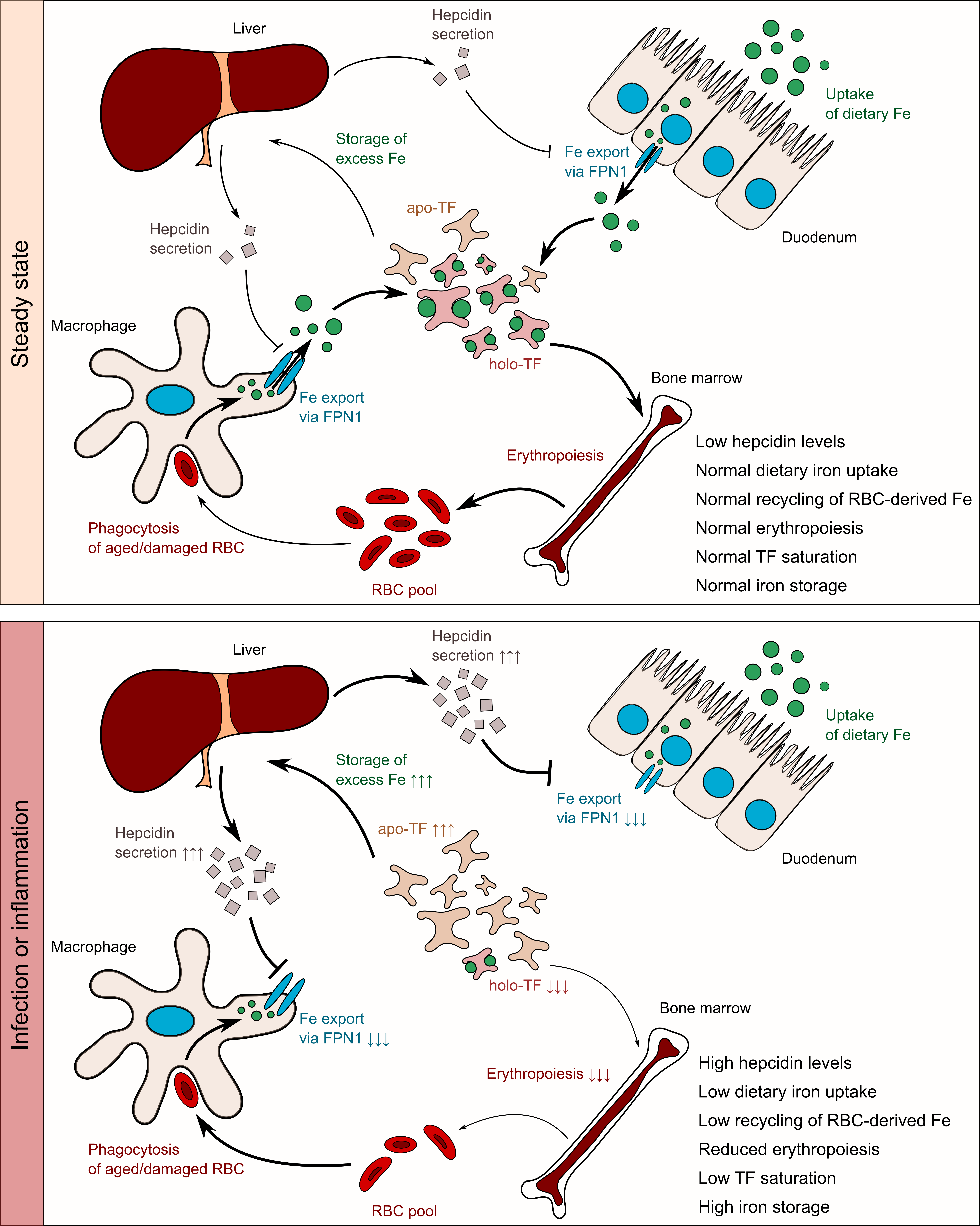

Supplement: Supplementary file 1 — Additional file 1. Overview of systemic iron turnover at steady state and during infection or inflammation. At steady-state (upper panel) levels of hepcidin, the inhibitor of ferroportin-1 (FPN1) – mediated cellular iron export, are low. This enables efficient dietary iron uptake in the duodenum and recycling of iron derived from aged and damaged red blood cells (RBC) in macrophages. As a result, iron saturation of circulating transferrin (TF) stays at physiological levels and sufficient iron is available for bone marrow erythropoiesis and other body organs. The excess iron is stored in the liver and spleen. During infection or inflammation (lower panel), hepcidin levels rise, hence blocking the FPN1-mediated dietary iron uptake and iron recycling in macrophages. Circulating iron is, in addition, extensively captured and stored by macrophages, the liver and spleen. Cumulatively, TF saturation decreases and iron availability for erythropoiesis, other cells of the body but also for potential pathogens such as bacteria, viruses or fungi is very low. If chronic, inflammation or infection may lead to reduced RBC hemoglobinization and cellularity manifested as anemia of chronic disease (ACD). [file 40560_2020_495_MOESM1_ESM.png]

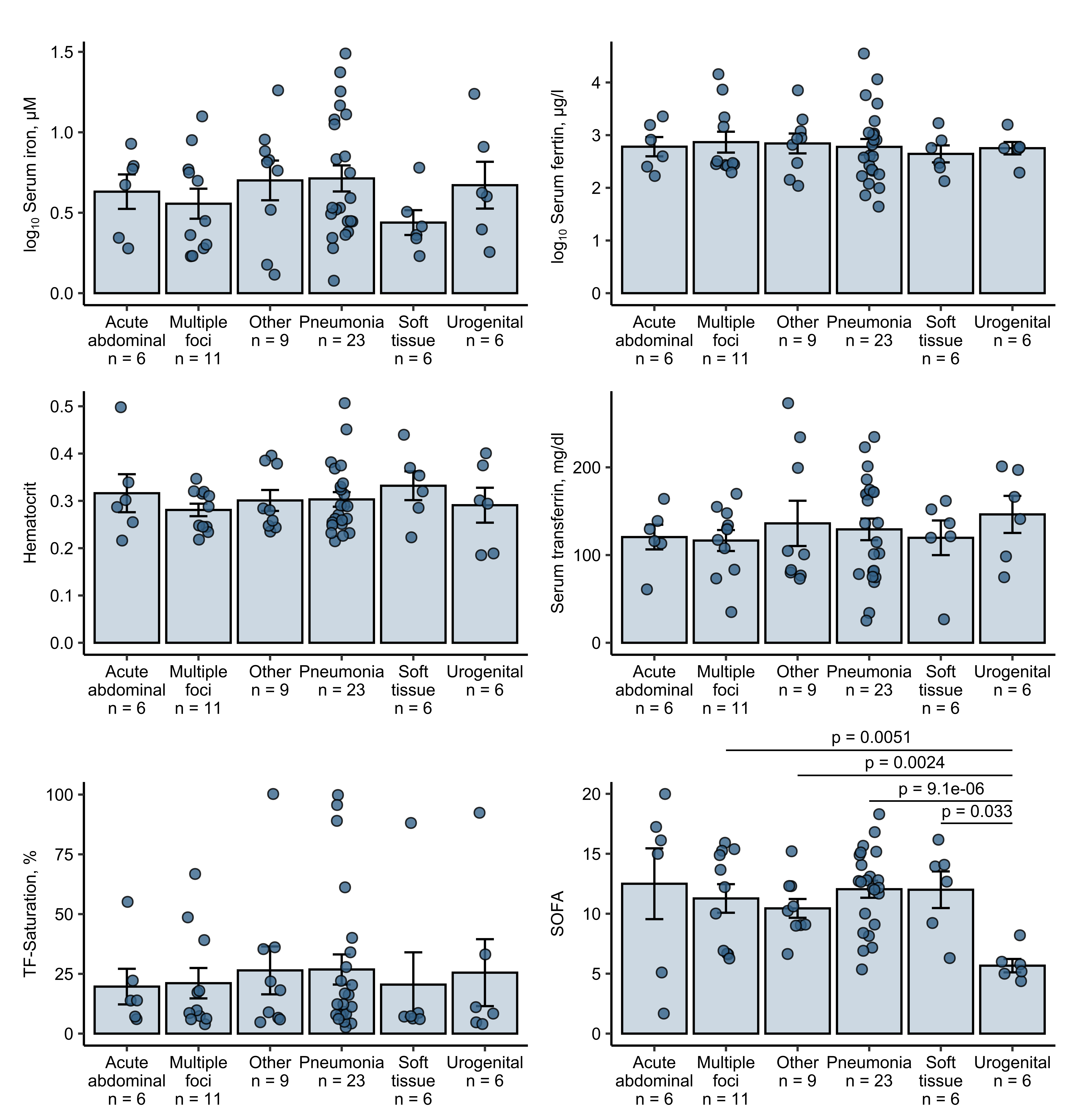

Supplement: Supplementary file 2 — Additional file 2. Regulation of iron turnover markers in study participants stratified by the primary infection foci. Each point represents a single observation, bars and whiskers depict mean with SEM. Statistical significance was determined with one-way ANOVA and Benjamini-Hochberg post-hoc two-tailed T tests. Strongly non-normally distributed ferritin and iron concentrations were log10 transformed. In the plots, n numbers of patients in each focus strata and significant post-hoc test p values are presented. ANOVA statistics: serum iron: F5, 55 = 0.81, ns; serum ferritin: F5, 55 = 0.12, ns; hematocrit: F5, 55 = 0.46, ns; transferrin: F5, 55 = 0.31, ns; transferrin saturation (TF-Sat): F5, 55 = 0.12, ns; SOFA: F5, 55 = 3.0, p = 0.018. [file 40560_2020_495_MOESM2_ESM.png]

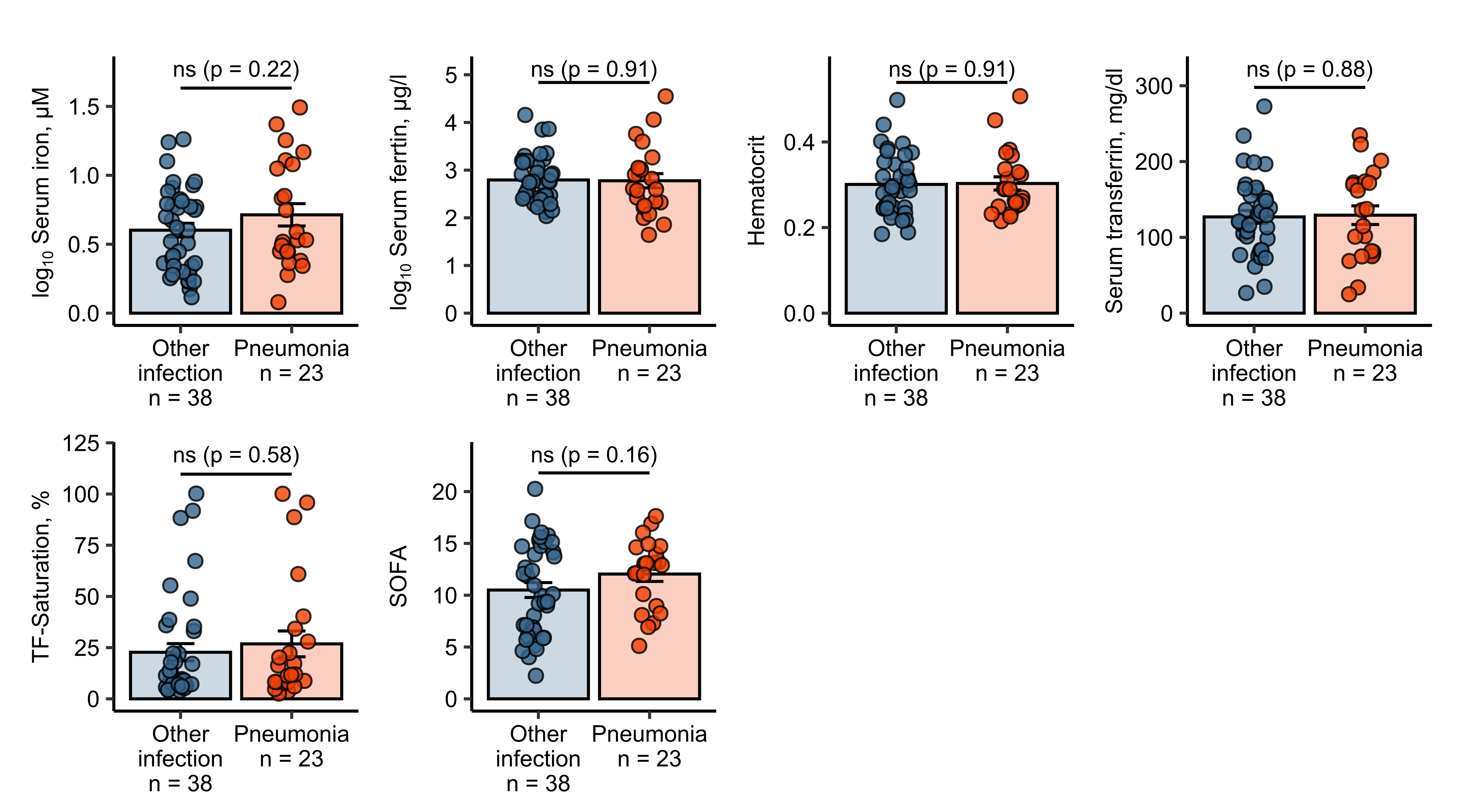

Supplement: Supplementary file 3 — Additional file 3. Regulation of iron turnover markers in study participant suffering from pneumonia and other infections. Regulation of iron turnover markers and SOFA in pneumonia patients and participants suffering from other infections. Each point represents a single observation, bars and whiskers depict mean with SEM. Statistical significance was determined with two-tailed T-test. Strongly non-normally distributed ferritin and iron concentrations were log10 transformed. In the plots, n numbers of pneumonia and other infection individuals and p values (two-tailed T test) are presented. [file 40560_2020_495_MOESM3_ESM.png]

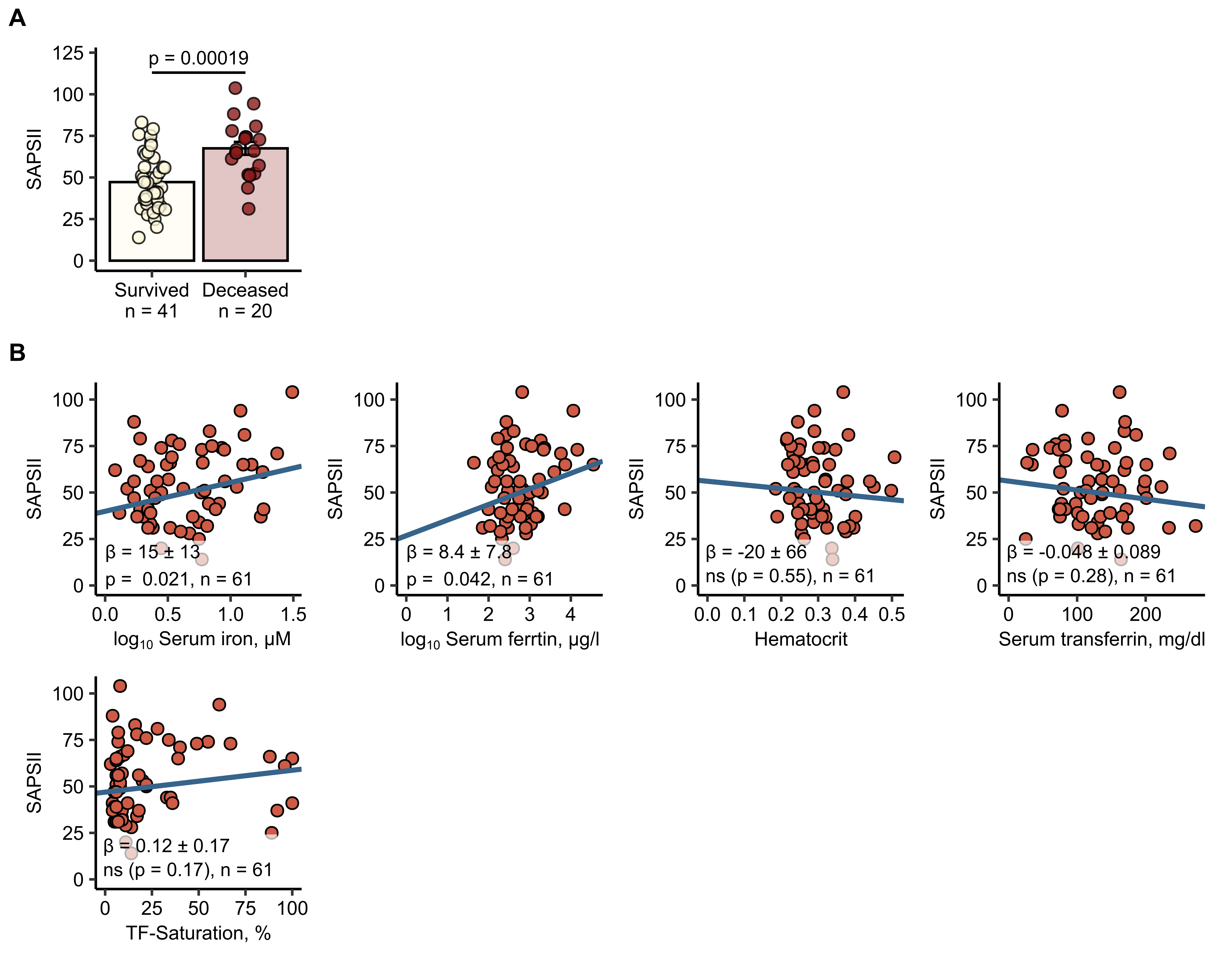

Supplement: Supplementary file 4 — Additional file 4. Correlation of iron turnover parameters with SAPS II. (A) Values of SAPS II in survivors and ICU deceased sepsis patients. Each point represents a single observation, bars and whiskers depict mean with SEM. Statistical significance was determined with mixed-effect linear model (fixed effect: vital status, random effects: gender and age). In the plot, n numbers of survivors and deceased participants and p value (two-tailed T test, β ≠ 0) are presented. (B) Correlation of iron parameters with SAPS II. SAPS II score value was modeled as a function of the iron turnover parameters with mixed-effect linear model (fixed effect: the investigated parameter, random effects: gender and age). For strongly non-normally distributed ferritin and iron concentrations log10 values were used for modeling. Each point represents a single observation, blue solid lines depict the fitted regression lines. Regression slope β estimates with 95% CI, p value for slope significance (two-tailed T test, β ≠ 0) and n numbers of observations are presented in the plots. [file 40560_2020_495_MOESM4_ESM.png]

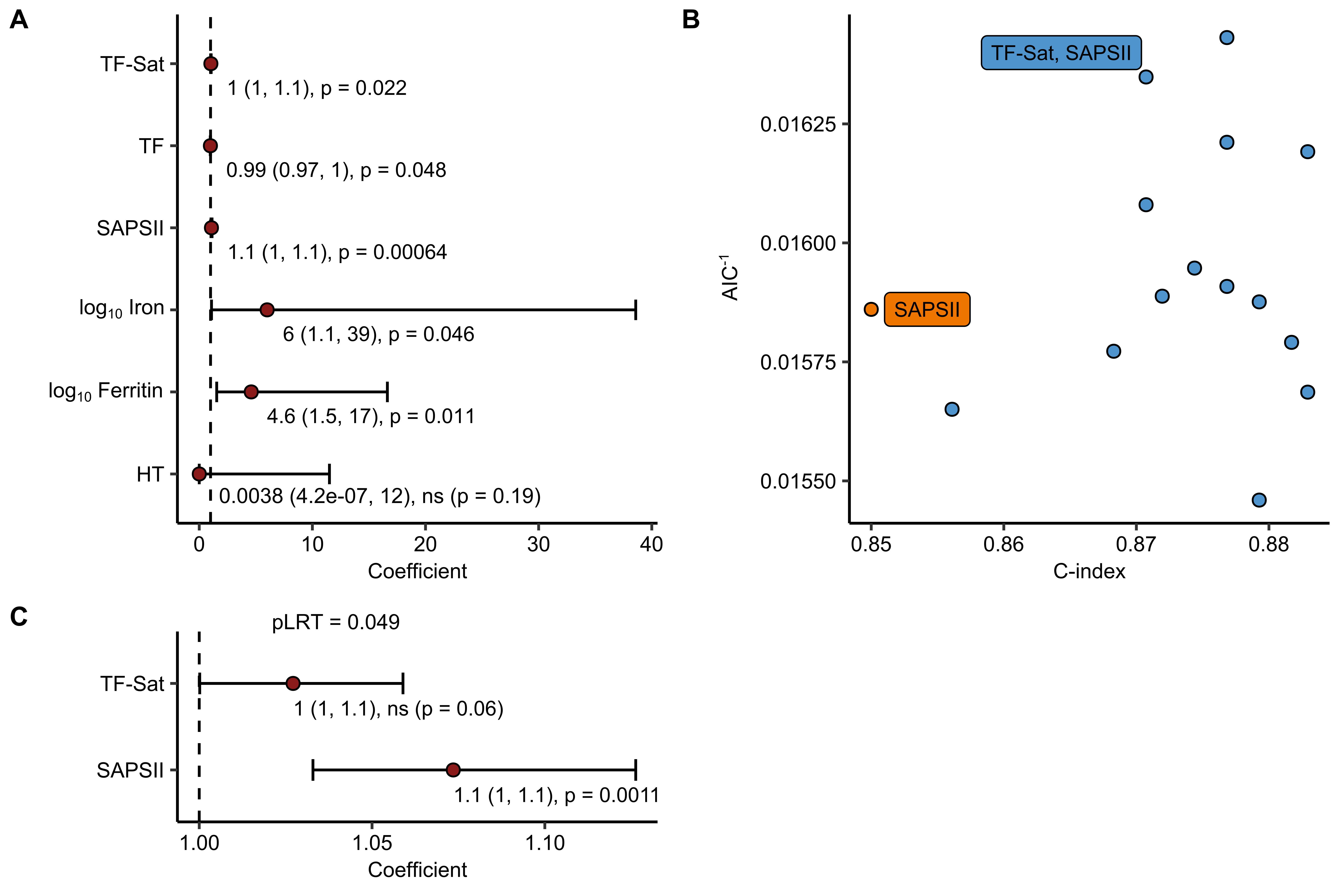

Supplement: Supplementary file 5 — Additional file 5. Dependence of transferrin saturation on SAPS II in predicting ICU survival. (A) Predictive power of age- and sex-corrected serum iron concentration, ferritin, transferrin (TF), TF saturation (TF-Sat), hematocrit (HT) and SAPS II. Correlation with ICU mortality for each of the investigated parameters was analyzed with a separate logistic regression model with inclusion of age and sex as confounders. Significance for regression β estimates was determined with Wald Z test. Results are presented as a forest plot. Points represent exponentiated β estimates, whiskers represent 95% CI. Points are labeled with exponentiated estimate, CI and p values. Survivors: n = 41, deceased: n = 20. (B) Comparison of predictive power for SAPS II and compound SAPS II-iron parameter models. A family of logistic regression models correlating SAPS II, 0 - 3 combinations of the iron/hematological parameters linked in (A) to survival as well as of age and sex as confounders with ICU mortality was generated. For each model, estimate p-values (determined with Wald Z test), Akaike Information Criterion (AIC) and concordance index (C-index) was calculated. The SAPS II-alone model and the SAPS II - TF-Sat compound model are labeled in the plot. Survivors: n = 41, deceased: n = 20. (C) Estimates for the SAPS II - TF-Sat compound model presented as a forest plot. Significance for regression β estimates was determined with Wald Z test. Points represent exponentiated β estimates, whiskers represent 95% CI. Points are labeled with exponentiated estimate, CI and p values. Significance of the inclusion of the TF-Sat term was assessed with likelihood ratio test (pLRT, compound model vs SAPS II-alone model). Survivors: n = 41, deceased: n = 20. [file 40560_2020_495_MOESM5_ESM.png]

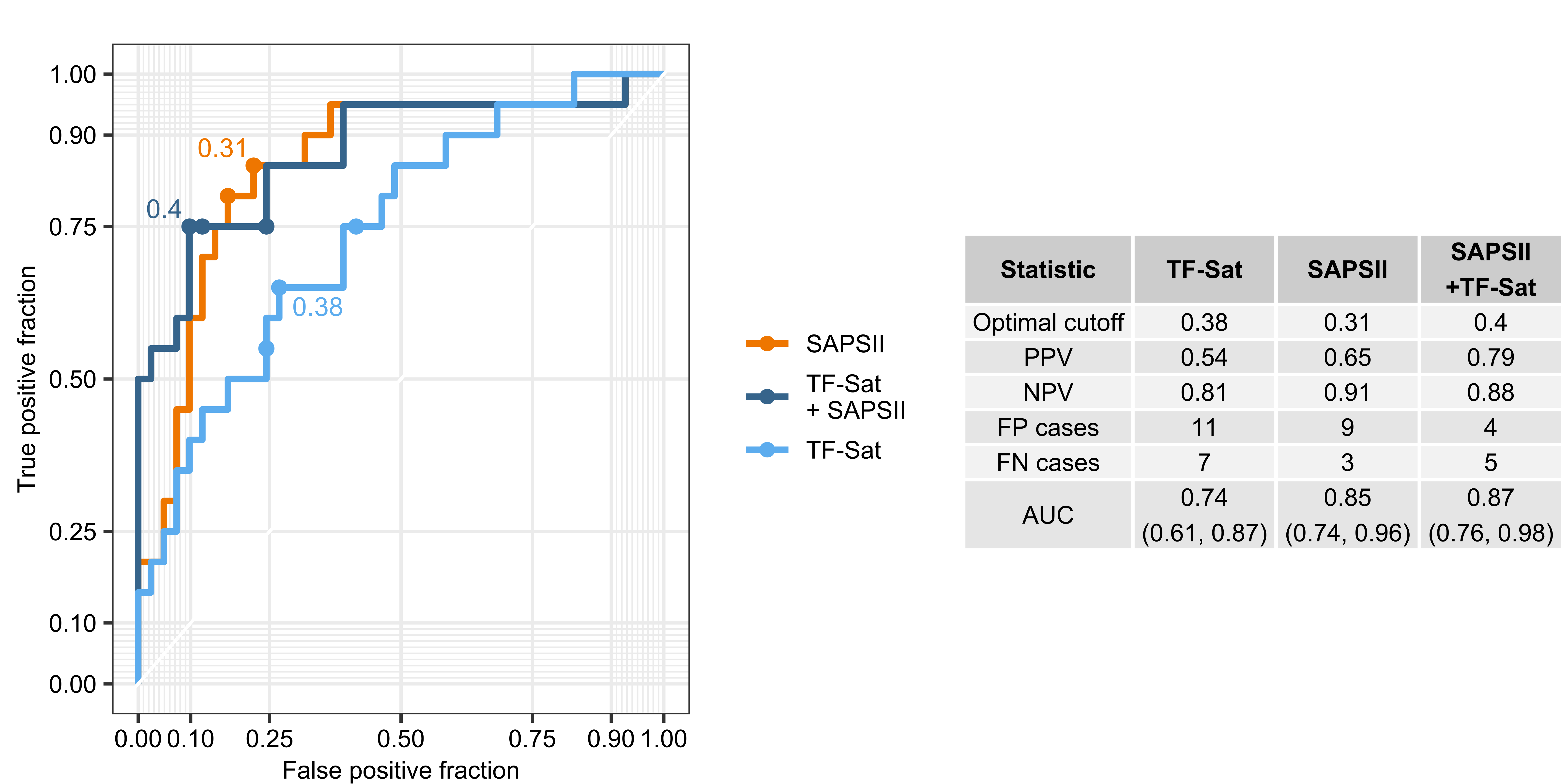

Supplement: Supplementary file 6 — Additional file 6. Predictive power of transferrin saturation in receiver-operator curve (ROC) analysis. Theoretical survival probabilities for study participants were calculated using the age- and sex-adjusted SAPS II, age- and sex-adjusted TF-Sat and the age- and sex-adjusted SAPS II compound model presented in Figure S5B (SAPS II - TF-Sat). The plot displays ROC for the models with optimal cutoffs labeled. The table presents calculated optimal cutoffs, positive and negative predictive values at the optimal cutoff (PPV and NPV), numbers of false-positive and false-negative cases at the optimal cutoff (FP and FN) and area under the curve (AUC) for ROCs with 95% CI. [file 40560_2020_495_MOESM6_ESM.png]

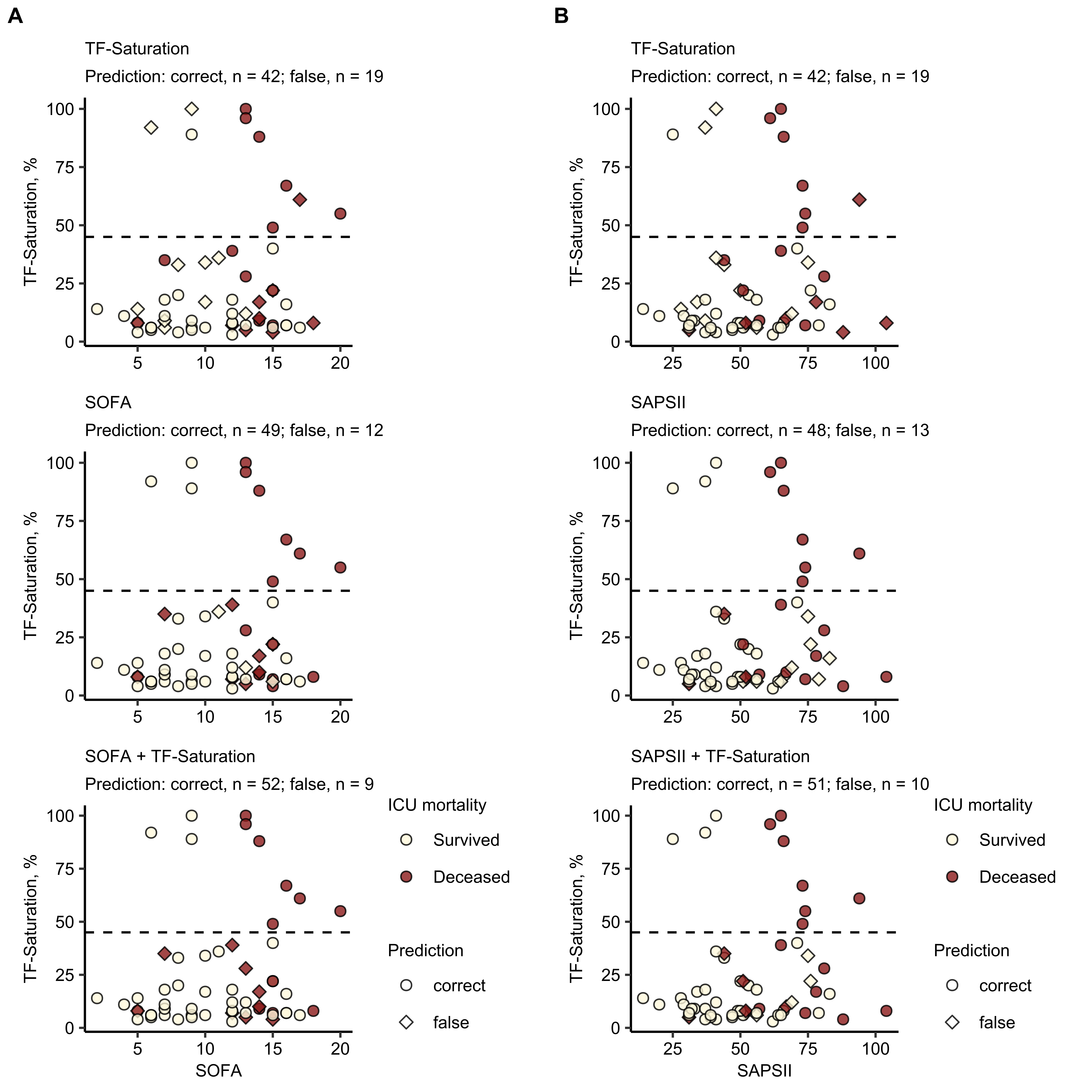

Supplement: Supplementary file 7 — Additional file 7. Accuracy of ICU mortality prediction by the SOFA - TF-Sat and SAPS II - TF-Sat compound models. Theoretical survival probabilities for study participants were calculated using (A) the age- and sex-adjusted SOFA, age- and sex-adjusted TF-Sat and the age- and sex-adjusted SOFA compound model presented in Figure S5B (SOFA + TF-Sat) and (B) the age- and sex-adjusted SAPS II, age- and sex-adjusted TF-Sat and the age- and sex-adjusted SAPS II compound model presented in Figure S5B (SAPS II + TF-Sat). For each model, optimal cutoffs were determined by ROC analysis as presented in Figure 3 and Figure S6 and patients were stratified into predicted survivors and predicted deceased and the predicted survival was compared with the actual ICU mortality (correct or false prediction). Point plots depict (A) TF-Sat and SOFA and (B) TF-Sat and SAPS II values, points fill codes for the actual vital status, point shape for quality of model prediction. Plots are tagged with the absolute numbers of correct and false survival predictions. [file 40560_2020_495_MOESM7_ESM.png]

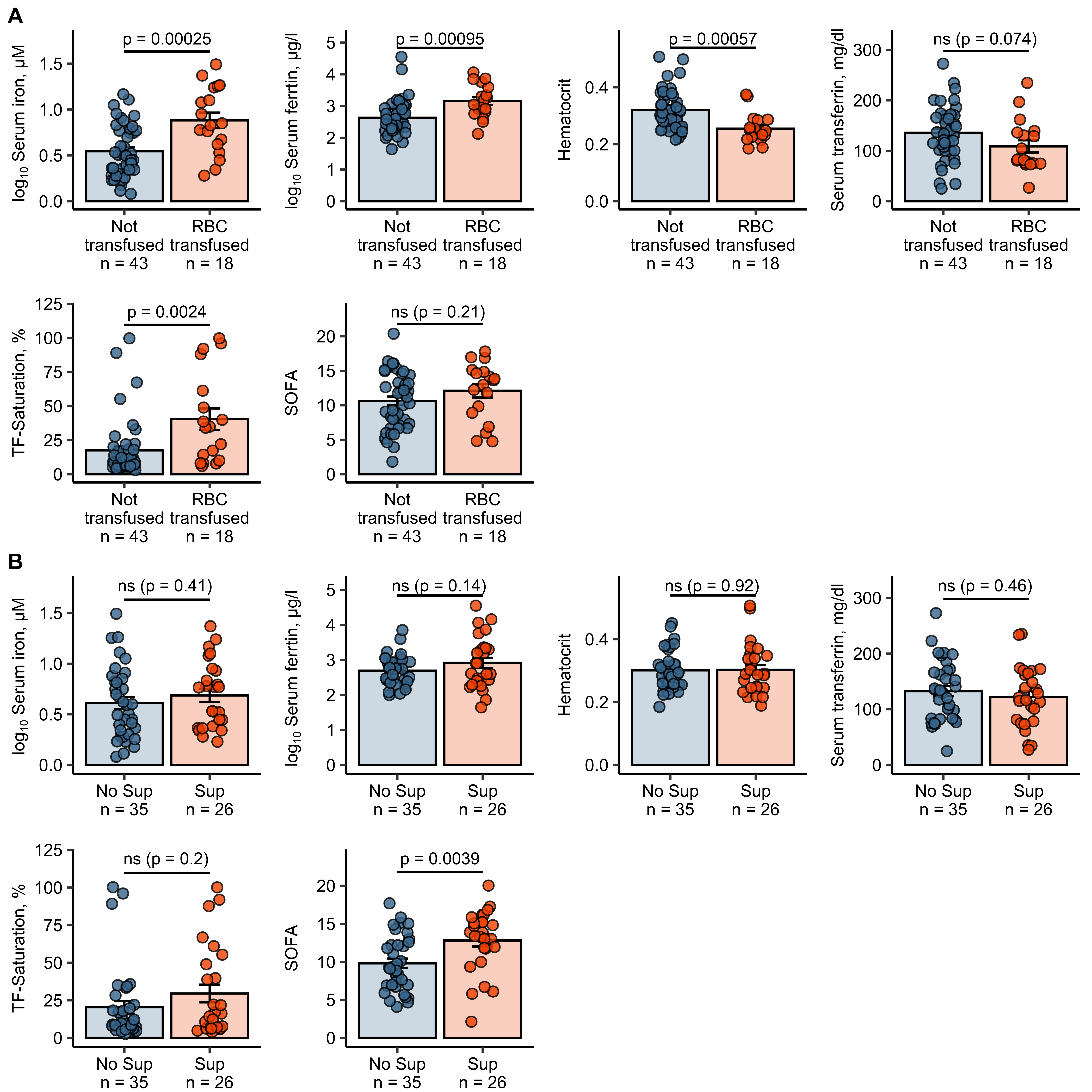

Supplement: Supplementary file 8 — Additional file 8. Regulation of iron turnover parameters in patients having been treated with RBC transfusions and immunosuppression before ICU admission. Study participants were stratified into the group who had been treated with red blood cell (RBC) transfusions or immunosuppressive co-medication within the last 3 months before ICU admission. (A) Regulation of iron parameters in RBC transfused and transfusion-naive study participants. Each point represents a single observation, bars and whiskers depict mean with SEM. Statistical significance was determined with two-tailed T-test. Strongly non-normally distributed ferritin and iron concentrations were log10 transformed. In the plots, n numbers of transfused and non-transfused individuals and p values (two-tailed T test) are presented. (B) Regulation of iron parameters markers in immunosuppression-treated (Sup) and immunosuppression-naive (No Sup) study participants. Each point represents a single observation, bars and whiskers depict mean with SEM. Statistical significance was determined with two-tailed T-test. Strongly non-normally distributed ferritin and iron concentrations were log10 transformed. In the plots, n numbers of immunosuppression-treated and -naive individuals and p values (two-tailed T test) are presented. [file 40560_2020_495_MOESM8_ESM.png]

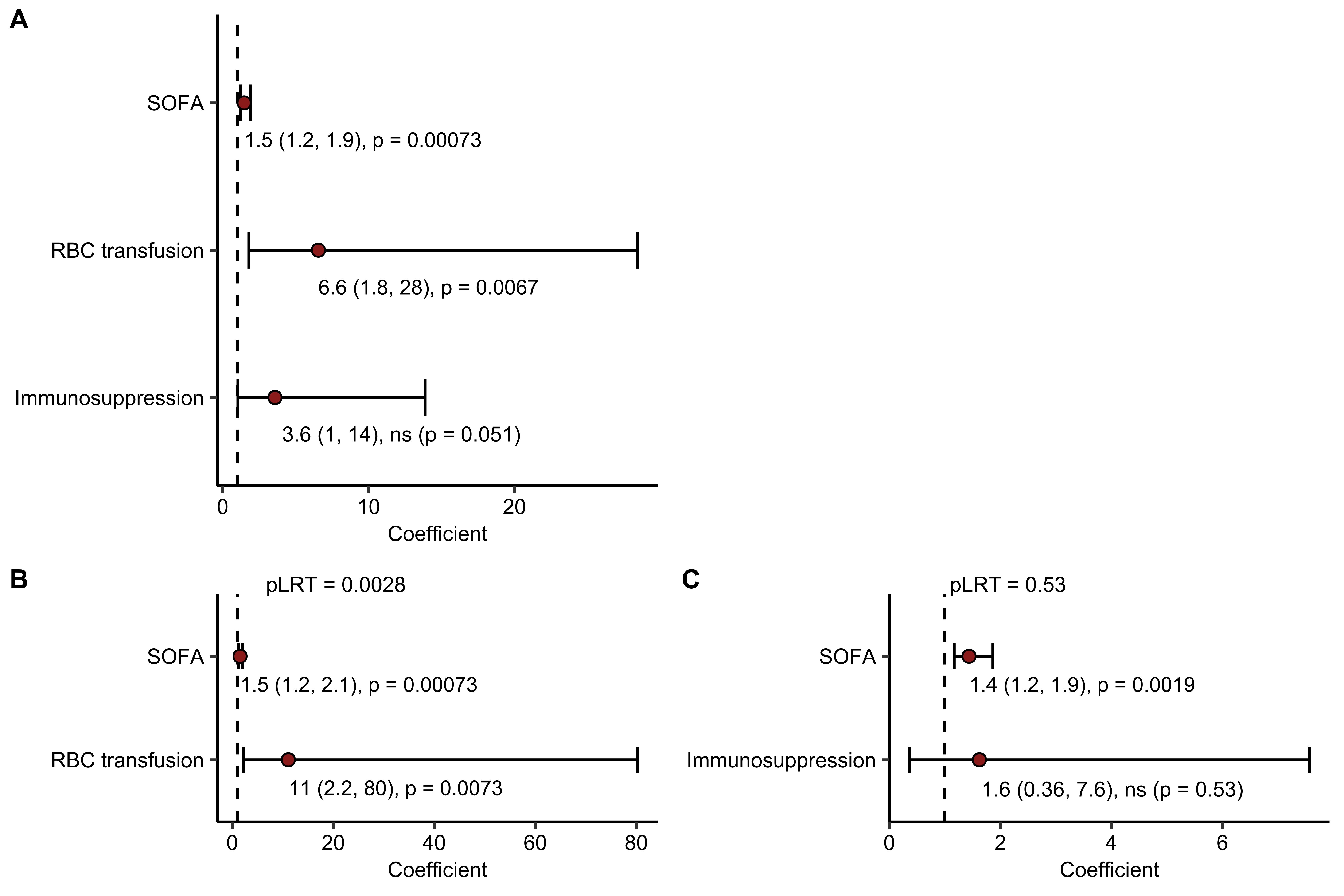

Supplement: Supplementary file 9 — Additional file 9. Predictive power and SOFA dependence of RBC transfusions and immunosuppression for ICU survival. Study participants were stratified into the group who had been treated with red blood cell (RBC) transfusions or immunosuppressive co-medication within the last 3 months before ICU admission. SOFA score was routinely determined at ICU admission. (A) Predictive power of age- and sex-corrected RBC transfusion, immunosuppressive therapy and SOFA. Correlation with ICU mortality for each of the investigated parameters was analyzed with a separate logistic regression model with inclusion of age and sex as confounders. Significance for regression β estimates was determined with Wald Z test. Results are presented as a forest plot. Points represent exponentiated β estimates, whiskers code for 95% CI. Points are labeled with exponentiated estimate, CI and p values. Survivors: n = 41, deceased: n = 20, RBC transfused n = 18, treated with immnosuppression: n = 26. (B, C) Predictive power of RBC transfusion- and immunosuppression-confounded SOFA score for ICU mortality. Correlations for SOFA and RBC transfusion status (B) as well as SOFA and immunosuppression (C) with ICU mortality were analyzed with separate logistic regression models with inclusion of age and sex as confounders. Significance for regression β estimates was determined with Wald Z test. Results are presented as a forest plot. Points represent exponentiated β estimates, whiskers represent 95% CI. Points are labeled with exponentiated estimate, CI and p values. Significance of the inclusion of the RBC transfusion and the immunosuppression term was assessed with likelihood ratio test (pLRT, SOFA - RBC transfusion vs SOFA and SOFA - immunosuppression vs SOFA). Survivors: n = 41, deceased: n = 20, RBC transfused n = 18, treated with immnosuppression: n = 26. [file 40560_2020_495_MOESM9_ESM.png]

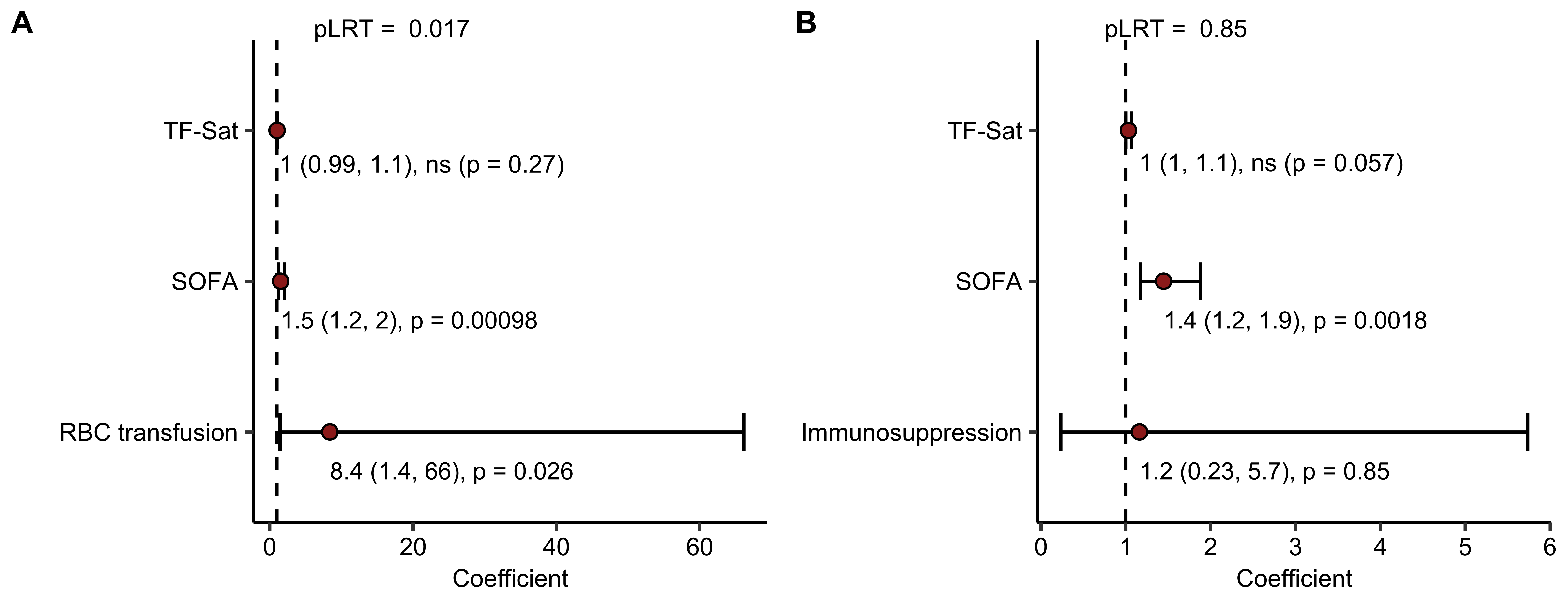

Supplement: Supplementary file 10 — Additional file 10. Effects of inclusion of the RBC and immunosuppression effects on the predictive value of the compound SOFA model. (A, B) Effects of inclusion of the RBC transfusion term (A) and the immunosuppression term (B) on the predictive value of the compound SOFA model. The best performing SOFA - TF-Sat compound model identified in Figure 2B was appended with terms coding for RBC transfusion status (A) and immunosuppressive medication (B). Significance for regression β estimates was determined with Wald Z test. Results are presented as a forest plot. Points represent exponentiated β estimates, whiskers represent 95% CI. Points are labeled with exponentiated estimate, CI and p values. Significance of the inclusion of the RBC transfusion and the immunosuppression term was assessed with likelihood ratio test (pLRT, SOFA - TF-Sat + RBC transfusion vs SOFA - TF-Sat, SOFA - TF-Sat + immunosuppression vs SOFA + TF-Sat). Survivors: n = 41, deceased: n = 20, RBC transfused n = 18, treated with immunosuppression: n = 25. [file 40560_2020_495_MOESM10_ESM.png]
